# Targeting allosteric binding site in methylenetetrahydrofolate dehydrogenase 2 (MTHFD2) to identify natural product inhibitors via structure-based computational approach

**DOI:** 10.1038/s41598-023-45175-3

**Published:** 2023-10-23

**Authors:** Nisarg Rana, Dhaval Patel, Meet Parmar, Nandini Mukherjee, Prakash C. Jha, Anu Manhas

**Affiliations:** 1 Department of Chemistry, School of Energy Technology, Pandit Deendayal Energy University, Gandhinagar, 382426 India; 2 Department of Industrial Biotechnology, Gujarat Biotechnology University, Gandhinagar, India; 3 School of Applied Material Sciences, Central University of Gujarat, Gandhinagar, 382030 India

**Keywords:** Computational biology and bioinformatics, Drug discovery, Molecular modelling

## Abstract

Cancer has been viewed as one of the deadliest diseases worldwide. Among various types of cancer, breast cancer is the most common type of cancer in women. Methylenetetrahydrofolate dehydrogenase 2 (MTHFD2) is a promising druggable target and is overexpressed in cancerous cells, like, breast cancer. We conducted structure-based modeling on the allosteric site of the enzyme. Targeting the allosteric site avoids the problem of drug resistance. Pharmacophore modeling, molecular docking, HYDE assessment, drug-likeness, ADMET predictions, simulations, and free-energy calculations were performed. The RMSD, RMSF, RoG, SASA, and Hydrogen-bonding studies showed that seven candidates displayed stable behaviour. As per the literature, average superimposed simulated structures revealed a similar protein conformational change in the αEʹ-βfʹ loop, causing its displacement away from the allosteric site. The MM-PBSA showed tight binding of six compounds with the allosteric pocket. The effect of inhibitors interacting in the allosteric site causes a decrease in the binding energy of J49 (active-site inhibitor), suggesting the effect of allosteric binding. The PCA and FEL analysis revealed the significance of the docked compounds in the stable behaviour of the complexes. The outcome can contribute to the development of potential natural products with drug-like properties that can inhibit the MTHFD2 enzyme.

## Introduction

Cancer is considered one of the prime causes of mortality worldwide. In 2022, it was observed that around 19.3 million new cases and 609,360 deaths were observed^1^. As per World Health Organization (WHO), the statistics of cancer deaths and new cases are predicted to reach 25 million over the next twenty years if not treated earlier^2^. Thus, based on cancer report-2022, cancer treatment should be prioritized^2^. In light of this, several anticancer drugs have been proposed. Besides, chemotherapy is also employed to fight cancer. However, the excessive use of chemotherapeutic drugs leads to the death of healthy normal cells, thus, causing severe side effects^3^. According to the WHO reports, chemotherapy in case of treatment of cancer is responsible for causing various side effects like instant toxicity and late chronic toxicity^4,5^. These toxicity symptoms can be life-threatening, severe, moderate or mild as defined by the WHO^4^. Instant effects are visible on hair, blood, skin, kidneys, gastrointestinal tract and bone marrow. Also, chemotherapy can affect various body organs like lungs, heart and brain^4^. Neurotoxicity caused by chemotherapy can induce weakness, paralysis, spasm, vomiting, fatigue, somnolence, hair loss and many more^4^. Apart from all these side effects, it also causes drug resistance, infertility and carcinogenicity^4^. Therefore, it is necessary to target the cancer-specific cells to deal with the severe cancer problem. In this regard, methylenetetrahydrofolate dehydrogenase 2 (MTHFD2) has gained attention as an attractive anticancer target due to its presence in the cancerous cells only^6^. It is reported that the level of MTHFD2 enzyme is increased in various types of cancers, developing embryos, and transformed cells, but its detection is low or minimal in the normal healthy cells^7^. Also, its detection enhances the risk of bladder cancer, hepatocellular carcinoma, colorectal cancer, and renal cell carcinoma. MTHFD2 is overexpressed in breast cancer, colorectal cancer, liver cancer, hepatocellular carcinoma, and bladder cancer too^7–9^. Along with this, studies show that the depletion of the MTHFD2 enzyme can cause cell death in various types of cancers^7^. Therefore, considering all these points, it can be concluded that the MTHFD2 enzyme is oncogenic and can act as a prognostic indicator, making it a promising therapeutic druggable target in cancer^7^. MTHFD2 plays a crucial role in performing one-carbon metabolism in human mitochondria, thus also known as NAD-dependent MTHFD-cyclohydrolase. It is involved in the biosynthesis of purines and thymidine building blocks^10^. This enzyme carries out the bifunctional activity; it catalyzes the dehydrogenation of 5,10-methylene-tetrahydrofolate in the presence of NAD + cofactor followed by the cyclohydrolysis of 5,10-methenyl-tetrahydrofolate to produce 10-formyl-tetrahydrofolate, thus results in the production of formate as a one-carbon unit^11^. MTHFD1 is known as the homologous protein of MTHFD2, and as per the Needle program in the European Molecular Biology Open Software Suite (EMBOSS), it shares 55.6% of sequence similarity with its homologous MTHFD2 enzyme^12^. Both of the enzymes are involved in carrying out the same reaction, but the only difference is that the MTHFD1 is expressed in normal cells too, thus; designing inhibitors against MTHFD2 specifically is expected to provide a wider therapeutic window with less toxicity and side effects^6^. MTHFD2 is a homodimer enzyme where each domain, i.e., N and C domains, forms a cleft of α/β strands. These domains are further connected by two α helices. These α/β strands contain highly conserved amino acids^13^. NAD + cofactor binds with one side of the strands, and the substrate-binding cavity is located at the interface of the two domains. As per the studies, MTHFD2 is involved in purine synthesis, and there are evidence that suggests the undefined role of the enzyme in carcinogenic transformation and embryonic development^10^. The role of the MTHFD2 enzyme in causing cancer is explained by its overexpression in the tumour cells and its relation with the patients suffering from cancer. Moreover, to explain the role of the MTHFD2 enzyme in causing cancer, gene knockdown studies have been reported that showed the impact of the MTHFD2 enzyme in causing depletion of the cancers^14^. Inhibition of the MTHFD2 enzyme reduces tumour growth, migration, invasion, and proliferation and promotes cell death, chemosensitivity and differentiation in various cancer cells^7^. Thus, the clinical importance of the MTHFD2 enzyme has intensified the interest of researchers in developing therapeutics against the MTHFD2 enzyme. In the literature, few inhibitors are reported against the MTHFD2 than the MTHFD1 enzyme. Gustafsson et. al. reported the first MTHFD2 inhibitor, a folate analogue LY345899. In the active site of the MTHFD2 enzyme, LY345899 showed interactions with crucial amino acids, like, Asn87, Lys88, Val131, Leu133, Asp155 and Gly310 and which possesses the capability to inhibit the enzyme with inhibitory activity of 663 nM (IC_50_). However, this inhibitor was proved to be a more potent inhibitor for MTHFD1 with IC_50_ of 96 nM^9^. The inhibitor LY345899 interacts in the cleft between the two lobes of the enzyme, also known as the substrate binding position, along with the NAD + cofactor^9^. Also, Chengzhang et. al. reported a natural substance, carolacton (inhibitor of E. coli isoform), to be active against MTHFD1 and MTHFD2 enzymes. However, the carolacton was selective towards MTHFD1 rather than MTHFD2 enzyme^15^. A new class of MTHFD2 inhibitors based on the caffeine scaffold was reported by Raze Therapeutics^16^. Also, several MTHFD2-specific inhibitors with tricyclic coumarin skeletons (sulphonamide derivatives) were reported by Kawai et. al., and from enzymatic assay studies, they observed the IC_50_ value as 1.6µM^17^. Also, these inhibitors showed hydrogen bond interaction with crucial amino acids like Asn87, Lys88, Gln132 and Gly310. However, these molecules lacked the potency against the MTHFD2 enzyme. In the complex, the inhibitors interact in the same substrate binding pocket but showed different binding modes compared to the LY345899^17^. Later, they discovered DS18561882, an MTHFD2 selective inhibitor that displayed the inhibitory activity of 6.3 nM (IC_50_ value)^18^. However, the compound still lacked potency; therefore, the same research group modified the chemical scaffold of the already reported inhibitor (DS18561882) to enhance the in vitro and in vivo efficacy^18^. Many inhibitors are reported against the enzyme; however, very few crystal structures of the MTHFD2-inhibitor complex have been reported. In most of the reported complexes, the inhibitors bind in the substrate binding cavity, thus, inhibiting the catalytic functioning of the enzyme. Therefore, in most of the studies, the interaction of the inhibitors is reported in the substrate-binding domain. Recently, a new allosteric binding site crystallised with xanthine derivatives was reported by Lee et. al., which is entirely distinct from the reported one (substrate-binding)^6^. Moreover, they have reported that the binding of the xanthine derivative in the allosteric site leads to the conformational change in the protein, which prevents the binding of the cofactor NAD + and phosphate to the enzyme^6^. Allosteric sites provide various routes for drug discovery, and the molecules that bind in the allosteric site are considered highly specific as they do not bind in the active cavity of the protein^19^. Furthermore, numerous studies have highlighted the advantages of allosteric inhibitors in drug development^20^. Allostery is a naturally occurring process which causes a conformational and functional change in the protein. The research has shifted to the search for inhibitors against the allosteric sites owing to the difficulty of drug resistance and the search for alternative inhibitors for the substrate binding site^19^. One key advantage is that the allosteric binding site typically exhibits lower conservation than the substrate-binding site among enzymes within the same family. Consequently, allosteric inhibitors tend to display higher levels of selectivity than nonallosteric inhibitors, resulting in fewer off-target side effects^21^. Finding inhibitors that form interaction within the allosteric site of the protein also reduces the chances of drug resistance and enhances their selectivity as well^19^. While searching for the studies reported on the allosteric site of the MTHFD2 protein in the literature, it was observed that until now, the work was limited to xanthine derivatives only. As per our understanding, this is the only study reported on the allosteric site with xanthine inhibitors. Also, the crystal structures of the MTHFD2 enzyme were reported with the inhibitors of the xanthine derivatives class only. The less exploration of the inhibitors against the allosteric site of the MTHFD2 enzyme gives an opportunity to explore the interaction of various inhibitors against the enzyme. Therefore, considering the importance of the MTHFD2 enzyme, allosteric site in the drug design, the lack of variety of inhibitors tested against this enzyme, and the limitation of the research conducted on this enzyme, we performed our studies on the allosteric site of the MTHFD2 enzyme using natural products. Despite recent work in the cancer treatment against the MTHFD2 enzyme, still limited wet lab and molecular modeling attempts have been made to discover the effective inhibitors against the allosteric site of the MTHFD2 enzyme, which explain the importance and novelty of the current work.We present the in silico drug designing methods where structure-based tools were compiled with the molecular dynamics and free energy calculations to search for the inhibitors that can bind in the allosteric binding domain of the MTHFD2 enzyme. Moreover, the MTHFD2 enzyme was considered with the active site-bound substrate analogue while performing all the calculations. As per our understanding, for the first time, the multicomplex-based pharmacophore modeling is performed on the allosteric bonded reported inhibitors of the MTHFD2 enzyme. The generated models were validated, and the representative pharmacophores were employed to conduct the virtual screening process using the natural product database. The screened allosteric candidates were compiled to undergo molecular docking followed by molecular dynamics simulations and free energy studies to scrutinise their binding stability within the biological conditions. Once computational studies shortlist an inhibitor, it should be procured or synthesized. The purified inhibitor should then be characterized for its physical and chemical properties, including solubility, lipophilicity and stability at various pH. Subsequently, in vitro and pre-clinical in vivo experiments are performed to understand its efficacy and toxicity profile (ADMET). Obtaining a crystal structure of the target protein-inhibitor complex (enzyme-inhibitor complex) remains one of the crucial steps in this context. Further in silico experiments can provide better insights with more concrete experimental evidence in hand^22,23^. Drug delivery is also crucial in drug discovery, where suitable excipients are to be determined and tested. It’s essential to analyse an inhibitor's actual in vivo performance before it is taken to clinical studies and considered therapeutic. The inhibitors retrieved in the studies can be used as potent anticancer compounds to conduct further drug-designing processes like in vitro studies and drug optimization. The synthesis of these compounds is under process, and after synthesis, they will be evaluated for their anticancer activities. As per literature, natural compounds are important for diagnosing and treating diseases, including cancer^24^. They can be multi-target specific and might affect multiple biological pathways simultaneously. There is both advantage and challenges to this aspect. As an advantage, the therapeutic window becomes broader if a compound is multi-target specific. Therefore the compound becomes particularly suitable for complex heterogeneous diseases like cancer^25,26^. On the other hand, the interaction of the compound with undesired biological pathways may lead to off-target effects leading to toxicity/side effects. It is therefore suggestive to experimentally determine the therapeutic/safety limitation (in vitro or in vivo) to choose lead based on optimum multi-target affinity to avoid side effects.

## Methodology

### Collection of the allosteric-bound protein complexes

From the RCSB database, all the reported complexes (allosteric sites) of the MTHFD2 enzyme were downloaded^27^. The selection of the MTHFD2 enzyme was based on the following criteria: (1) the crystallised protein–ligand complex must have experimentally reported inhibitory activity, (2) if the same protein–ligand complex is crystallised at two different resolution values, the one with higher resolution must be selected, (3) the inhibitors must be co-crystallised in the allosteric site of the enzyme (Table 1)^28^. Finally, the selected enzymes were prepared separately in the Discovery Studio suite version 4.0 via *Prepare Protein* protocol^29^. The preparation of the protein involves the steps of the elimination of crystallographic water, removal of alternate conformation, addition of missing atoms and loops, protonation of the titrable residues at physiological pH, and assigning the acid dissociation constant. Further, the prepared proteins were superimposed via *Align and Superimposed Protein* module of Discovery Studio suit of version 4.0^30^ to generate a single coordinate file.

**Table 1:** List of the allosteric-bonded PDBs of MTHFD2 protein selected for conducting the superimposition process.

PDB ID	Ligand ID	Resolution (Å)	Activity (IC_50_ μM)	Reference
7EHV	J4L*, J49	2.61	4.00	6
7EHN	J4F*, J49	2.25	0.69	6
7EHM**	J4C*, J49	2.13	0.78	6

*Inhibitors binded in the allosteric site of MTHFD2 protein (selected for superimposition).

**Selected as reference.

The main aim of constructing the superimposed coordinate file is to generate a common coordinate file encompassing all the reported allosteric site inhibitors and their crucial interactions responsible for showing inhibition of the enzyme. Based on the resolution value, apart from the reference protein chain, all other chains were deleted from the single coordinate file, thus, leaving the coordinates of one protein chain and the superimposed inhibitors bound in the allosteric site. The main aim of deleting the protein chain was to avoid the repetition of the same MTHFD2 chain. The generated coordinate file was subjected to common feature pharmacophore modeling generation.

### Common feature pharmacophore generation

The single coordinate file was subjected to common feature pharmacophore generation by using the *Common Feature Pharmacophore Generation* protocol of the Discovery Studio suit of version 4.0^30^. During pharmacophore preparation, the conformation generation of the cocrystallised ligands was disabled to retain the bioactive conformation for pharmacophore construction, as reported in our previous studies^28^. Also, the *HipHop* algorithm was implemented to construct the model at default parameters. During pharmacophore generation, the common features were obtained via the in-built *LUDI* program. The features were hydrogen bond acceptor (A), hydrogen bond donor (D), hydrophobic (H), hydrophobic aromatic (Y), hydrophobic aliphatic (Z), positive ionisable (P), negative ionisable (N), and ring aromatic (R).

#### Validation of pharmacophore models

The generated models were evaluated via a series of validation studies to search for the pharmacophores that can predict/screen their actives from a dataset with both actives and inactive ligands. The models were subjected to the preliminary test set validation method. Thereafter, the shortlisted hypotheses were subjected to an external validation process, i.e., Gunner Henry (GH) and Enrichment Factor (EF), to obtain an efficient model for the virtual screening (VS) process. External validation involves constructing a dataset with decoys seeded with known active ligands. Mainly, the quality of the constructed models can be evaluated by statistical parameters, viz. (1) specificity, which defines the capacity to exclude the inactive molecules, (2) sensitivity, which defines the capacity to screen the active compounds, (3) the yield of actives, which defines the ratio between the true positive and number of screened hits, (4) EF which explains the yield of actives in comparison to the composition of the screened dataset, (5) the GH scoring method, that calculate the percentage of yield of actives (EF), (6) percentage of sensitivity, which evaluate the efficiency of the screening of the dataset^31^. The value of GH varies from 0 to 1, where 1 represents the ideal value of the ideal mode. The generated ROC curves display the enrichment power of a model in plotting sensitivity against 1-specificity. The AUC display the performance of the pharmacophores and is considered a useful parameter in evaluating the model performance. The value of AUC also varies from 0 to 1. The value of 1 represents the ideal model where the actives are detected first in comparison to the inactive ligands, 0.5 represents the random screening result, and 0 represents the screening of inactive ligands prior to actives^31^.

To conduct the test set validation process, three separate datasets of 62 actives, 221 presumed inactive and 3895 presumed inactive ligands, were collected from literature and Decoy Finder^32^, respectively. These datasets were employed to conduct two sets of calculations. In the first set of calculations, 62 actives and 221 presumed inactive ligands were selected, whereas in the second set of calculations, 62 actives and 3895 presumed inactive ligands were considered. Owing to the lack of inhibitors of the allosteric site, we selected the inhibitors of the active site of the MTHFD2 enzyme as the active inhibitors. For presumed inactive, we selected ChEMBL database^33^ to retrieve presumed inactive by using the *Molecular Descriptor* based parameter of Decoy Finder^32^. From the test set validation parameter, we obtain parameters like specificity, sensitivity, and area under the curve of receiver operator characteristic (AUC-ROC). In general, all those models that fit the criteria (high specificity and sensitivity) were selected for conducting the virtual screening or secondary validation method. However, in the current study, we selected the models mainly based on their higher sensitivity and lower specificity value owing to the non-availability of the allosteric bonded inhibitors. The selected hypotheses were subjected to a secondary validation method. A mixed dataset of 15 actives and 796 presumed actives was used to conduct the secondary validation study. The mixed dataset was prepared as per the FAST *Conformation Generation* method under CHARMm force field^34^ of *Prepare Ligand* protocol of Discovery Studio version 4.0^30^. For the conformation generation step, maximum conformations were fixed to 255 with a minimum energy of 20.0 kcal/mol threshold. The Ligand Pharmacophore Mapping module was employed to conduct the secondary validation method. From the secondary validation method, we retrieve parameters like % yield of actives (A), % ratio of actives (RA), Goodness of hits (GH), and Enrichment factor (EF) score. Generally, the models shortlisted to conduct the VS should possess higher A, RA, GH and EF values. However, in our case, we utilise the dataset of actives prepared from the inhibitors bound in the primary binding site. Therefore, we may not obtain the expected values. Lesser A, RA, GH, and EF values will define our model's sensitivity.

### Database preparation and virtual screening

For conducting the virtual screening process, we selected natural product-based datasets. Extensive research has been conducted on natural products to check their activity against various cancer cell lines^35^. Also, as an advantage, they played a significant role in developing chemotherapeutic drugs, alone or in combination with other medicines, thus, can be used as a starting lead for various novel anti-cancer drugs^36^. Many hits are discovered from natural products that paved paths for developing new and effective anti-cancer agents. In drug design, more than 50% of FDA-approved drugs belong to the category of natural products or their derivatives. The source of natural products can be a chemically synthetic molecule synthesized based on the natural product, semi-synthetic natural product, and original natural compound^37^. Natural product molecules are essential for diagnosing and treating diseases, including cancer^24^. As per the studies conducted by Khazir et. al., natural products-based synthetic drug molecules account for more than 65%, and most of the plant-based natural products account for more than 75% of anti-cancer drugs^38^. Natural products are considered reducing agents, free radical scavengers, and singlet oxygen quenchers as they are rich sources of natural antioxidants. Their antioxidant property is due to bioactive compounds like isoflavones, isocatechin, flavonoids, and many more. They can reduce the toxic side effect of the chemotherapy treatments^36^. Therefore, considering the importance of natural products in the anti-cancer drug design, we selected three different open-access natural product datasets, i.e., Specs^39^, COlleCtion of Open Natural prodUcTs (COCONUT)^40^, Universal Natural Product Database (UNPD)^41^, and one in-built dataset of Discovery Studio version 4.0, i.e., DruglikeDiverse dataset (DDD). As discussed, natural products have gained attention from researchers as they play a significant role in drug discovery and have displayed selectivity towards the cellular targets^42^. Specs natural product database incorporates industrial catalogues. It is one of the datasets that include the catalogue of the chemical compounds synthesised and isolated via companies. They contain their chemical structure and annotations^39^. COCONUT is the dataset that includes unique natural product datasets of plant origin (phytochemicals), marine-based, and microbial-based origin. This dataset is available freely for conducting screening and other computational drug design applications^40^. UNPD dataset is a rich source of natural product structures that contain information on the chirality of the natural compounds, which can act as an advantage. UNPD database was built to compile all the natural product scaffolds in one dataset, which can be employed for in silico drug designing process^41^. The UNPD dataset is not available in the link in the actual publication; however, the structures are compiled and maintained by ISDB website^18^. Specs dataset had 813 molecules, COCONUT had 1000 molecules, UNPD possessed 2,29,358 natural products, and DDD possessed 5390 molecules. Overall, we selected 2,36,561 compounds for conducting a virtual screening process over the representative pharmacophores. The *Search 3D Database* module of the Discovery Studio version 4.0 was used to conduct virtual screening. However, before screening, the three open-access datasets (Specs, COCONUT, and UNPD) were built via the *Build 3D Database* module based on *the Catalyst* algorithm of the Discovery Studio version 4.0^30^. This method uses CHARMm force field^34^ to remove the structural duplicates, correct the atom types and bond types, add hydrogen atoms, and assign formal charges while generating 3D conformations based on the BEST *Conformational Generation* method with a flexible fitting mode. Finally, the screened candidates were saved in a single mol2 coordinate file, which was subjected to molecular docking studies.

### Allosteric site-based molecular docking and binding affinity calculations

All the screened candidates were docked in the allosteric site of the MTHFD2 protein (co-crystallised with the active site-bound substrate analogue). Docking calculations were performed using *FlexX* suit^43^ of LeadIT 2.3.2 software, a comprehensive drug design suit (BioSolveIT, GmbH)^44^. This suit uses incremental construction (IC) based algorithm to bind the ligand in the selected binding cavity. This algorithm constructs the molecule in three steps, i.e., selection of base, placement of base, and construction of the complex. During docking, the IC algorithm disintegrates the molecule into smaller moieties and then constructs it incrementally within the selected binding domain of the protein, thus providing flexibility simultaneously^43^. Also, the module uses a modified Böhm scoring function to calculate the binding free energy (ΔG) of the protein–ligand interactions of the docked complexes^45^.

$$\Delta G={\Delta G}_{o}+ {\Delta G}_{rot}+ {N}_{rot}+ {\Delta G}_{hb} \sum_{\begin{array}{c}neutral \\ H-bond\end{array}}f\left(\Delta R, \Delta \propto \right)+ {\Delta G}_{io}\sum_{\begin{array}{c}ionic \\ interactions\end{array}}f\left(\Delta R, \Delta \propto \right)+ {\Delta G}_{aro}\sum_{\begin{array}{c}aromatic \\ interactions\end{array}}f\left(\Delta R, \Delta \propto \right)+ {\Delta G}_{lipo}\sum_{lipo contact}{f}^{*}\left(\Delta R\right)$$

In the above equation, ΔG_o_ represents the fixed ground term of the protein–ligand complex, ΔG_rot_ and N_rot_ represent the conformational entropy caused by the binding of the ligand, ΔG_hb,_ ΔG_io,_ and ΔG_aro_ calculate the pairwise interactions based on the geometrical interaction model function *f*, ΔR represents the distance conditions, and Δα represents the angular conditions, the last term in the modified Böhm scoring equation represents the hydrophobic interactions and unfavourable steric clashes because of atom–atom interactions^43,44^.

Prior to the molecular docking, redocking was performed on J4C to validate the docking software and protocol applied in the current study. Thereafter, molecular docking calculations were conducted on 7EHM allosteric site by using screened natural product compounds. We selected the allosteric site as it is reported in the literature that identifying allosteric inhibitors confirms more selectivity than the non-allosteric site inhibitors^21^. Also, targeting the allosteric site avoids the drug resistance issue, which arises mainly because of mutation in the active binding site to the protein^6,21^. Based on the resolution value, we selected 7EHM as the reference PDB co-crystallised with xanthine derivative (J4C) as the reference inhibitor for docking calculations. To perform docking, the protein chain was prepared using the *Receptor Preparation* tool of the LeadIT 2.3.2 program. This step ensures the assigning of atom types, the addition of polar hydrogen atoms, removal of crystallographic water. The default allosteric binding site of 6.5 Å was defined from the centre of the co-crystallised inhibitor JC4. The selected docking site incorporates all the crucial residues that form bonding with the inhibitors and are responsible for causing inhibition. The chemical ambiguities existing in the allosteric sites were resolved by *ProToss* module of the LeadIT 2.3.2 program^46^. This module optimises the reference ligand (JC4), co-factor (if any), and amino acids and assigns the protonation state and tautomers to the allosteric binding cavity of the protein. Default docking and chemical parameters were employed to deal with the steric clashes that arise within the protein–ligand binding cavity. Moreover, an enthalpy and entropy-based docking scheme was used to place the disintegrated fragments into the binding cavity of the protein. All the successfully docked candidates were subjected to HYDE assessment to check their binding affinity within the allosteric binding site^47^. This module helps in investigating the stability of the docked complexes by integrating the factors like desolvation effects, hydrogen bonding, and hydrophobic effect during complex formation. After HYDE assessment, based on the ligand binding affinity, all the selected candidates were subjected to drug-likeness and ADMET studies. The docked protein–ligand 2D-interaction plots were constructed by using the PoseView module of the LeadIT 2.3.2 program^48^.

### Drug-likeness and pharmacokinetic properties

All the prioritised HYDE-assessed candidates were subjected to Drug-likeness studies as the primary filter, followed by ADMET studies as the secondary filter. Drug-likeness studies were conducted using Lipinski’s rule of five^49^ and Veber's rule^50^. For the primary filter, eight descriptors were calculated, i.e., number of hydrogen bond acceptor, donor, molecular weight, lipophilicity (AlogP), polar surface area, number of aromatic rings, number of rotatable bonds, and the total of hydrogen bond donor and acceptor. These filter parameters were computed via the *Filter Ligand* protocol of Discovery Studio version 4.0^30^. In the current work, the natural product database was selected; therefore, considering the nature of the database, the number of violations allowed was kept at ‘1’^51^. As per the criteria, only those molecules were selected for the ADMET studies that possess molecular weight < 500 Dalton, hydrogen bond donor, and AlogP < 5, hydrogen bond acceptor < 10 as per Lipinski’s rule of five^49^, and the number of rotatable bonds < 10, polar surface area < 140 Å^2^, and the number of rotatable bonds < 10 as per Veber rule^50^. After primary filtration, the filtered compounds were subjected to ADMET studies. For secondary filtration, six descriptors were calculated, i.e., blood–brain barrier (BBB), plasma protein binding (PPB), hepatotoxicity, aqueous solubility, intestine absorption, and CYP2D6 binding. To perform secondary validation, *Calculate Molecular Property* module of Discovery Studio version 4.0^30^ was utilised. Different values of the ADMET parameters explain different behaviour of the molecules. In the case of solubility level, 0 defines extremely low solubility, 1 defines very low but possible solubility, 2 defines low, 3 defines good solubility, 4 defines optimal, and 5 defines too soluble. In the case of ADMET BBB level, 0 defines high penetrating power, 2 defines medium penetration, 3 defines low penetration, and 4 remains undefined. An adsorption level of 0 defines good absorption, 1 defines moderate absorption, and 2, and 3 define poor and very poor absorption respectively. As per ADMET cut-off criteria, only those candidates were selected that showed FALSE prediction for CYP2D6 and Hepatoxicity, TRUE prediction for PPB, favourable values of solubility level (3), adsorption level (0), and BBB level (2 ad 3). The filtered candidates were evaluated via their 2D-interaction plot retrieved via molecular docking studies. Based on the presence of crucial interactions, the final shortlisted candidates were subjected to molecular dynamics simulation studies.

### Molecular dynamics (MD) simulation of MTHFD2 and MTHFD2 docked complexes

Classical MD simulations were performed for the Methylenetetrahydrofolate dehydrogenase (MTHFD2) apo-protein, MTHFD2_reference inhibitor (J4C), and MTHFD2 in complex with selected docked compounds. A total of 9 MD systems were built and subjected to a 300 ns simulation run. All the docked complexes that were subjected to MD had two ligands: one crystallographic ligand (J4C) docked to the main site and other docked compounds bound to the allosteric site. As performed in our previously reported studies^52–54^, MD simulations were undertaken using GROMACS ver.2020.6^55^ with the CHARMM 36 force field^56^. Compounds parameters and topologies were generated using the CGenff server^57^. All MD simulation systems were solvated using the spc216 water model with a dodecahedron box configuration and a 1 nm distance from the protein's edges in all directions. The MD systems were then neutralised with an equal number of counter ions (Na^+^/Cl^−^), and further energy minimization with the steepest descent algorithm was done to eliminate the steric collisions, poor contacts and generate a maximum force of less than 1000 kJmol^−1^ nm^−1^ (50,000 steps max). Post energy minimization, position restraint equilibration was performed for 1 ns under NVT (constant number [N], constant volume [V], and constant temperature [T]) and NPT (constant number [N], constant pressure [P], and constant temperature [T]) ensembles. The Berendsen thermostat algorithm^58^ was employed in NVT equilibration to keep the system at a constant volume (100 ps) and temperature (300 K). Furthermore, NPT equilibration was performed at a constant pressure (1 bar) for 100 ps using the Parrinello-Rahmanbarostat^59^. The Particle Mesh Ewald approximation was employed with a 1 nm cutoff to calculate long-range electrostatic interactions and coulombic and Van der Waals interactions^60^. The LINCS algorithm^61^ was used to set a restriction on the length of the bonds. Finally, a 300 ns simulation run with the default parameters was performed, and coordinates were saved every 2 fs. The choice of simulation time and convergence criteria aims to strike a balance between capturing relevant dynamics and obtaining accurate and reliable results while efficiently using computational resources. The MD simulations were conducted for the time duration of 300 ns, as influenced by the biological questions being addressed for MTHFD2 protein as mentioned in previous studies^62,63^. VMD^64^ and UCSF Chimera were used to visualise the MD simulation trajectories^65^. GROMACS' built-in 'gmx' commands were used to calculate root mean square deviation (RMSD), root mean square fluctuation (RMSF), and hydrogen bonds (H-bonds), among other properties, and the plotting tool GRACE was used to generate and visualise the plots, as reported in our previous studies^66,67^.

### Computation of binding free energy using MM/PBSA

The Poisson-Boltzmann or generalised Born and surface area continuum solvation (MM/PBSA and MM/GBSA) are conventional and well-acknowledged approaches for determining protein-inhibitor affinity. The MD trajectories of apo-MTHFD2, MTHFD2_JC4 and MTHFD2_docked complexes were utilized for computing binding free energies. The binding free energy calculations and energy contributions from individual residues were utilised to quantify the compound affinity for MTHFD2. In general, the binding free energy of the protein–ligand complex is expressed as the difference in the free energy of the complex and the total free energy of the isolated ligand and protein. The g_mmpbsa tool^68^ with default parameters was used to calculate the molecular mechanics potential energy (E_MM_) (electrostatic + Van der Waals interactions) and solvation-free energy (polar + non-polar solvation energies).

$$ \begin{gathered} {\text{E}}_{{{\text{MM}}}} = {\text{ E}}_{{{\text{bonded}}}} + {\text{ E}}_{{{\text{nonbonded}}}} \hfill \\ {\text{E}}_{{{\text{nonbonded}}}} = {\text{ E}}_{{{\text{vdW}}}} + {\text{ E}}_{{{\text{elec}}}} \hfill \\ \end{gathered} $$

E_bonded_ represents the interactions due to bond, angle, dihedral and improper interactions, E_nonbonded_ encompasses both E_vdW_ (van der Waals) and E_elec_ (electrostatic) interactions. E_vdW_ interactions are modelled by Lennard Jones (LJ) potential function, and E_elec_ interactions are modelled using Coulomb potential function. As per studies, in the single trajectory, the protein–ligand conformation in the bound and unbound states are considered to be the same. Therefore, E_bonded_ is considered zero.

$$ {\text{G}}_{{{\text{solvation}}}} = {\text{ G}}_{{{\text{polar}}}} + {\text{ G}}_{{{\text{nonpolar}}}} $$

G_polar_ represents the electrostatic contribution, and G_nonpolar_ represents non-electrostatic contributions to the solvation-free energy (G_solvation_).

The binding free energy of both molecules was calculated, i.e., for docked compounds in the allosteric site and crystallographic ligands in the main site. The stable 200 ns (1000 frames) trajectories from each bound complex, as determined by the RMSD plot, were used to estimate binding free energy. The frames were selected at a regular interval of 200 ps covering a broad range of trajectories to cover various conformational spaces of the docked complexes for improved structure–function correlation. As discussed, the MM/PBSA method is commonly used to estimate binding free energies in molecular dynamics simulations. However, it comes with limitations and assumptions. The PBSA component employs an implicit solvent model, which might not fully capture solvent effects for charged or polar surfaces and can be influenced by dielectric constant choices. Its accuracy is reliant on force field parameter quality. Thus, we have used uniform dielectric constant values and the same force field for all the simulation setups. Accurate binding free energy calculations require thorough conformational sampling, but MM/PBSA's reliance on a single MD trajectory might lead to incomplete energy landscape exploration. Therefore, we extracted the last stable and converged trajectory of 200 ns for conducting the calculations. The method's partitioning into polar and nonpolar contributions assumes additivity, potentially oversimplifying intricate binding interactions. The Poisson-Boltzmann equation assumption in MM/PBSA neglects polarization effects, which can be significant in binding events.

## Results and discussion

### Common feature pharmacophore generation

**Figure 1:**
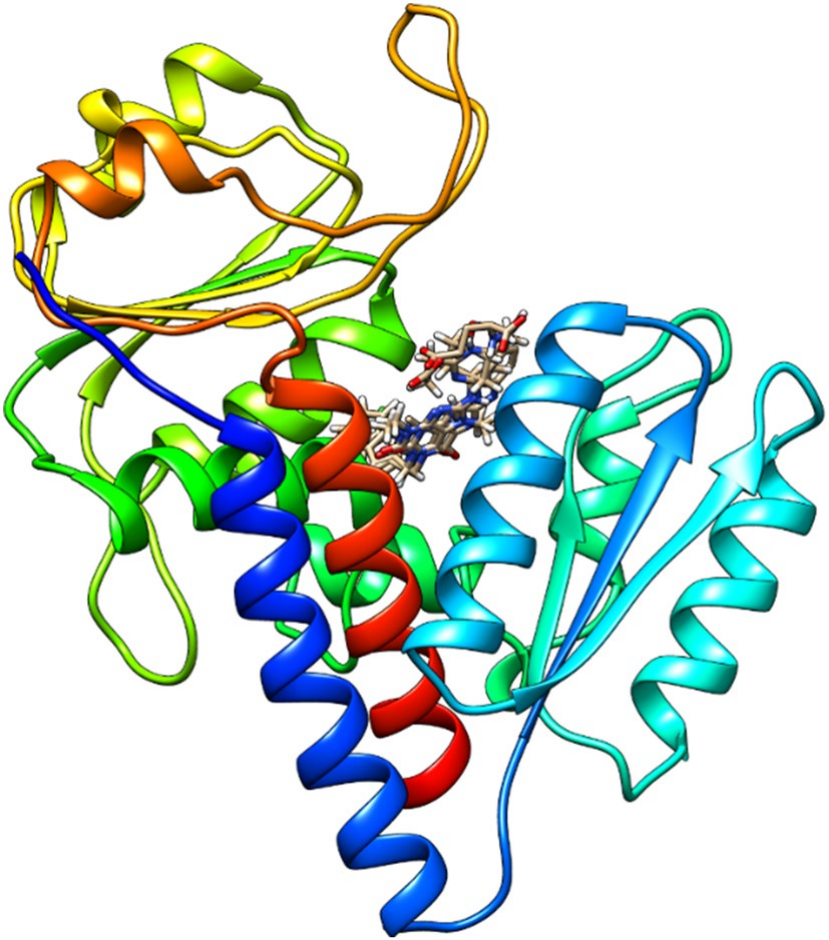
Pictorial representation of the superimposed coordinates of the allosteric-bonded inhibitors of MTHFD2 protein.

The superimposed coordinates of the PDBs of the MTHFD2 enzyme were used to generate the pharmacophore models (Fig. 1). As discussed, during pharmacophore generation, the conformation generation of the superimposed coordinates of inhibitors was disabled to recognize the crucial experimental interactions only. Essential interaction features like A, D, H, Y, Z, P, N, and R were selected during the process of pharmacophore construction. These features are considered essential for an inhibitor to form an interaction with the amino acids of the protein chain. Ten models were selected from the Maximum Pharmacophore generation option. To study the close chemical interactions, Minimum Interfeature Distance was selected as 2 Å. Other parameters like the Number of Leads That May Miss and Feature Misses were set to 1 to construct the models that may consider the molecules that did not possess all the features required for the pharmacophore construction. Furthermore, to search for the molecules that may not map to any feature of the generated models, the option of Complete Misses was selected as 0. With the help of the in-built LUDI program, the protein–ligand interactions were converted into Catalyst supported features like A, D, H, Y, Z, P, N, and R. A sum of ten common feature models were generated (Table 2) via *Common Feature Pharmacophore Generation* protocol of Discovery Studio v 4.0^30^.

**Table 2:** List of ten generated pharmacophore features along with the pictorial representation and difference in spatial orientation in similar feature models like models 2 and 3, models 5 and 6, and models 8, 9 and 10.

Model	Features	Pictorial representation	Difference in spatial orientation
01	YZZAA		YZZAA
02	RZZAA		
03	RZZAA		
04	YZZHD		YZZHD
05	RZZHD		
06	RZZHD		
07	ZZHHA		ZZHHA
08	YZZHA		
09	YZZHA		
10	YZZHA		

On observing the generated features from the superimposed protein–ligand complexes, it is evident that all the constructed models contain five features. The ten different pharmacophores generated display different combinations of features defining the interactions formed within the protein–ligand complex. Model 1 contains YZZAA, Model 2 and 3 contain RZZAA, Model 4 contain YZZHD, Model 5 and 6 contain RZZHD, Model 7 contain ZZHHA, and Model 8, 9 and 10 contain YZZHA chemical features. Among the generated five features, hydrophobic aliphatic (Z) interaction is commonly observed, representing the common hydrophobic aliphatic, an essential intermolecular interaction between the inhibitors and protein (Table 2). This hydrophobic aliphatic (Z) feature is formed due to the halogen-substituted aromatic ring that occupies the hydrophobic pocket of the MTHFD2 enzyme. The aromatic ring, which is halogen-substituted, also forms π-Sulphur interaction with Met165, π-Sigma interaction with Val162 and Pro208 residues of the allosteric site of the protein. Also, the xanthine moiety of the inhibitors is responsible for showing π-π interactions with the amino acids Phe157. On observing the models having similar combinations of chemical features, it can be observed that the spatial orientation of one or more chemical features is different (Table 2). The inter-feature distance within the features in the constructed models is shown in Supplementary Table S1.

In models two and three, the common five features (RZZAA) were retrieved. Though the features were common, but, the spatial orientation of the ring aromatic group (R) was different in both sets (Table 2). Similarly, the difference in the spatial orientation of the ring aromatic group (R) was observed in the case of models five and six, where common RZZHD features were obtained (Table 2). In the last three models, i.e., models eight, nine, and ten, common YZZHA features were obtained. Models eight and nine differ in the orientation of the hydrophobic group (H), whereas model ten differs via the spatial orientation of the hydrogen bond acceptor group (A) (Table 2). The fact that these chemical features are present in all the active compounds demonstrates the significance of these features. Moreover, these features represent the interactions responsible for showing inhibition of the MTHFD2 enzyme.

#### Pharmacophore validation

The generated hypotheses were subjected to a series of validation processes, viz. primary validation test set method, external validation GH and EF studies. As discussed, the quality of the constructed models was evaluated by parameters, viz. specificity, sensitivity, ROC and AUC values, EF, and GH scoring, yield of actives, and percentage of sensitivity. Once the pharmacophore models are developed, validation is crucial to evaluate their efficiency in predicting the actives and inactive during the VS process. In general, to conduct the validation study, two separate datasets of actives and inactive are generated, or a single dataset of inactive seeded with actives is prepared. In our case, there is a deficiency of the actives of allosteric sites of the MTHFD2 enzyme. Therefore, due to the deficiency of the allosteric site inhibitors, we collected all the active inhibitors of the substrate binding site of the MTHFD2 enzymes.

Primarily, the models were screened via the test set validation method. In the test set validation method, specificity defines the capacity of the model to screen out actives only during the validation process. Sensitivity determines the capacity of the model to discard the presumed inactive during the validation process. AUC-ROC defines the ability of the model to screen out actives before presumed inactive ligands. To conduct the test set validation method, 62 actives (active site bound) and 221 presumed inactive were collected as two different datasets. These inactives were retrieved from the Decoy Finder via a Molecular Descriptor-based parameter. The outcome of the test set method is shown in Supplementary Table S2. From the outcome, we can observe that all the models displayed higher specificity (> 0.99). This means that our models can predict the true negatives from the respective dataset. It is visible in the test set outcome that all models predict more than 219 molecules to be inactive out of 221 presumed inactive. However, in the case of specificity, the models displayed less ability to predict the active molecules. Most of the models screened a maximum of up to 20 compounds as actives out of a dataset of 62 actives. Thus, these models displayed sensitivity in the range of 0.08–0.32. In general, an ideal pharmacophore model displays a sensitivity and specificity value near to 1. However, in our case, our models were predicted to show lower sensitivity due to the lack of the actives of the allosteric binding site. Owing to the shortage of allosteric site-bonded inhibitors, the main objective of the validation process in the present work was to check the predictive ability of the model to exclude the actives (substrate-binding site) and presumed inactive ligands. Less value of sensitivity and false negative defines the sensitivity of our model. Interestingly, by looking at the scenario, we can predict that all the models have the predictive ability to differentiate the inactive. Lesser sensitivity is obtained because of the lack of allosteric site-binding inhibitors. In other words, we can say that our models can discard the presumed inactive ligands, even from a dataset made of active site-bonded inhibitors of the MTHFD2 enzyme. Thus, higher specificity and lower sensitivity represent the high efficiency of the models in excluding the inactive ligands. Also, this can be related to the difference in the interacting chemical groups in the active and allosteric binding sites of the MTHFD2 enzyme. To further validate our test set outcome, we conducted primary studies using the same 62 actives (active site bound), but this time, we collected 3895 diverse presumed inactive ligands. A similar trend of results was obtained from the outcome of the second test set method, as shown in Supplementary Table S2. The retrieved models displayed lower sensitivity and higher specificity defining the high efficiency of the models in excluding the inactive ligands. Based on the outcome, all the models were considered for the external validation process, i.e., GH and EF methods.

For conducting ligand mapping in secondary evaluation, we prepared a dataset of 796 molecules which contain 15 actives and 781 presumed inactive. Similar to the outcome in the test set method, it was observed that in GH and EF methods, all the models displayed less yield and ratio of actives, which reveals the lower accuracy of the models in recalling actives from the mixed dataset (Supplementary Table S3). The probable reason for the lower accuracy is the deficiency of the actives of the allosteric site of the MTHFD2 protein. The effect of the lower percentage of yield and ratio can be easily seen in the EF and GH scores of the models. It is obvious that the models do not consider selected hits (15) as their actives which leads to lower accuracy and lower reliability (Supplementary Table S3). However, this behaviour was observed due to the selection of actives of the active site of the MTHFD2 protein. Based on the test set primary validation, EF, and GH scoring external validation, we selected all models for conducting the virtual screening process.

### Common feature pharmacophore-based virtual screening

As all the models were shortlisted for conducting the virtual screening process, we retrieved a total of 1221 molecules (Supplementary Table S4). It is observed that model 1 screens out 195 candidates (18 COCONUT, 64 DDD, 35 Specs, and 78 UNPD). Similarly, models 2 to 10 screen out 108 (13 COCONUT, 40 DDD, 18 Specs, and 37 UNPD), 206 (19 COCONUT, 64 DDD, 25 Specs, and 90 UNPD), 32 (08 COCONUT, 03 DDD, 10 Specs, and 11 UNPD), 73 (09 COCONUT, 07 DDD, 08 Specs, and 49 UNPD), 92 (10 COCONUT, 18 DDD, 11 Specs, and 53 UNPD), 252 (132 COCONUT, 07 DDD, 60 Specs, and 53 UNPD), 95 (16 COCONUT, 22 DDD, 26 Specs, and 31 UNPD), 94 (15 COCONUT, 23 DDD, 25 Specs, and 31 UNPD) and 74 (17 COCONUT, 17 DDD, 20 Specs, and 20 UNPD) natural product molecules. The compiled dataset of 1221 molecules was prepared using *Prepare Ligand* protocol of Discovery Studio v 4.0^30^. As we were screening common datasets over ten different models, there is a probability of retrieving the same compounds. Therefore, the retrieved sets were prepared to discard the structural duplicates. After preparing the dataset, we retrieve 494 unique natural product compounds. This set of 494 molecules was utilised to conduct the molecular docking study.

### Molecular docking and binding affinity calculations

All the screened unique natural compounds were docked in the allosteric binding site of the MTHFD2 protein. Prior to the molecular docking, we performed redocking calculations. For redocking, based on the resolution value (2.13 Å), we selected 7EHM PDB ID. The PDB 7EHM contain two co-crystallised inhibitors, i.e., J49 and J4C. The J49 is co-crystallised in the active binding site, and J4C is co-crystallised in the allosteric binding site of the protein chain (Supplementary Fig. S2). Redocking calculations were conducted on J4C, and it was observed that the redocked pose was generated with an RMSD value of 1.5 Å, and it displayed all the crucial interactions with the amino acids Glu141, and Arg142, as reported in the experimental pose (Supplementary Fig. S3). Thus, the presence of all crucial interactions, as reported in the experiment, validates our software. Thereafter, all the 494 molecules were docked within the allosteric binding site of the protein, having crystallised J4C inhibitor. It was observed that, out of 494, 155 were not docked, and 339 natural product molecules were able to show binding in the allosteric binding site of the MTHFD2 protein (Supplementary Table S5). These 339 molecules were further assessed by HYDE calculations. This calculation helps to check the inhibitor's affinity within the protein cavity. HYDE assessment incorporates the factors like desolvation effects, hydrogen bonding, and the hydrophobic effect observed during the complex formation. The outcome showed that out of 339 molecules, 72 displayed better binding efficiency (Supplementary Table S5). These 72 molecules were subjected to drug-likeness and pharmacokinetic studies.

### Drug-likeness and pharmacokinetic properties

Drug-likeness and ADMET studies were conducted on all 72 molecules with an aim to eliminate the molecules with undesirable physicochemical, pharmacokinetic and toxicity properties. Of 72 molecules, 63 displayed an acceptable range of drug-likeness properties (Lipinski’s rule of 5 and Veber’s rule). Further, their ADMET properties were studied. Out of 63 shortlisted candidates, only 20 molecules displayed an acceptable range of parameters, as shown in the Supplementary Table S6. The 2D interaction plots generated from the 20 shortlisted docked compounds were checked. Out of 20, only 7 displayed the interaction with the essential amino acids of the allosteric binding site (Supplementary Tables S6 and S7).

### 2D-interaction analysis of the reference compound JC4 and shortlisted candidates

**Figure 2:**
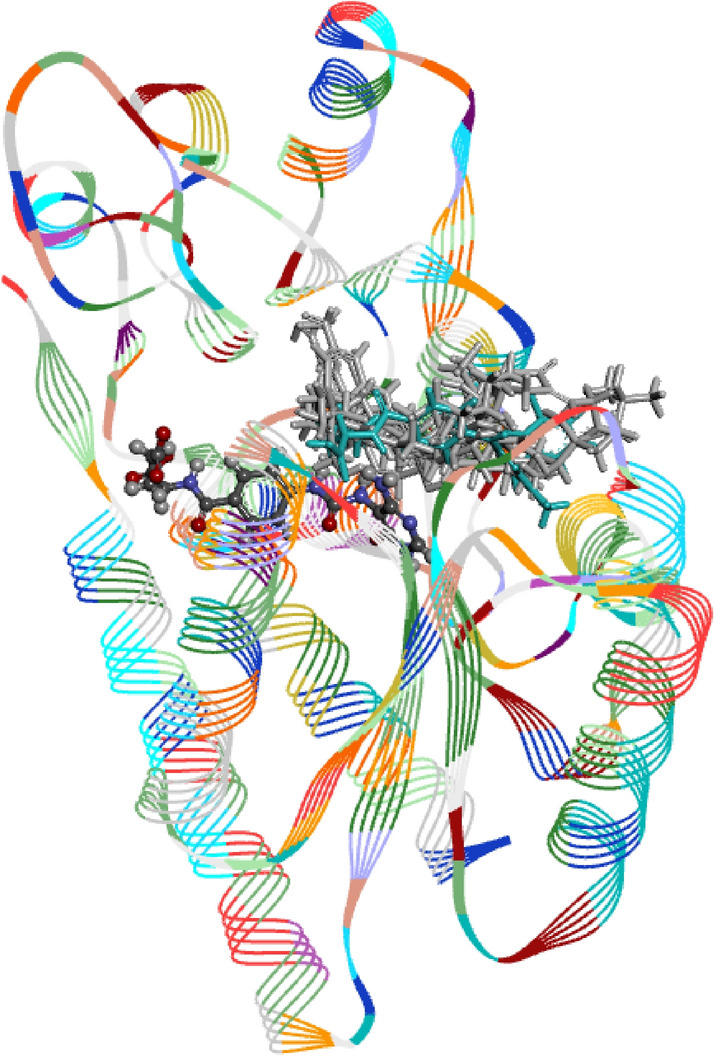
Pictorial representation of 3D-coordinates of docked poses (light grey), along with reference-JC4 (light blue) and active site-bound inhibitor-J49 (brown) in the MTHFD2 protein.

**Figure 3:**
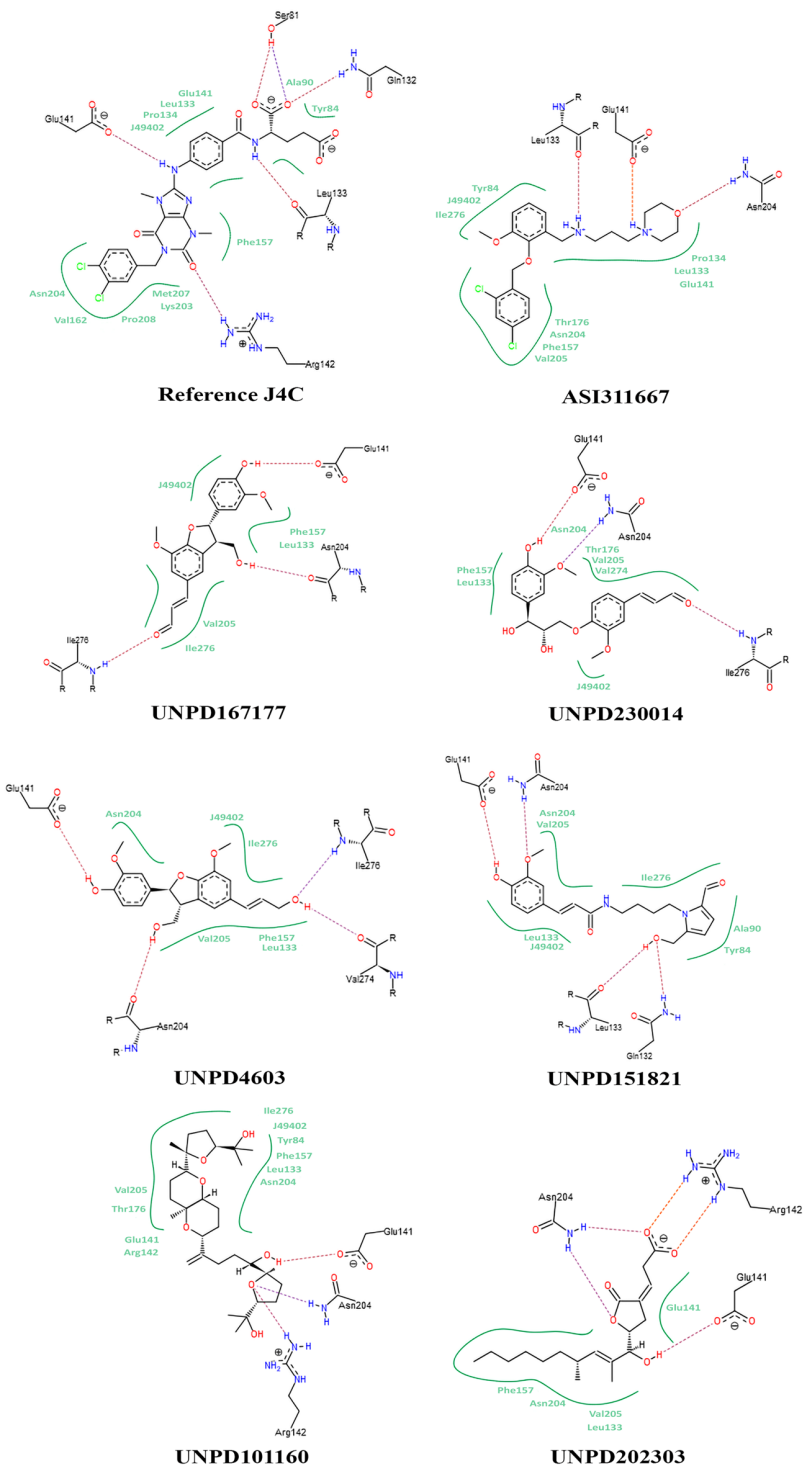
Pictorial representation of 2D-interaction plots of seven shortlisted docked natural products in the allosteric site of MTHFD2 protein.

Figures 2 and 3 explain the interactions observed in the allosteric binding site of the MTHFD2 protein. On observing the docked poses of seven shortlisted candidates (Fig. 2), it is obvious that all the molecules occupied the allosteric binding domain of the protein.

As per the enzymatic studies reported in the literature, the hydrogen bond interaction with the glutamic acid (Glu141) and asparagine (Asn204) of the allosteric site of the enzyme plays a crucial role in causing the inhibition of the enzyme^6^. In the case of top scored candidate, ASI311667, the oxygen atom of 4-methylene morpholine moiety forms a hydrogen bond with the side chain of Asn204, also the –NH group of the same moiety forms a hydrogen bond interaction with the side chain of Glu141 (Fig. 3). The 2,4 dichloro-1-(2-methoxy-phenoxy-methyl) benzene group of the top-scored candidate was observed to form hydrophobic contacts with the protein chain and act as a hydrophobic head group. The remaining part of the molecule, N-methylhexanamine, form interaction via the –NH group with the side chain of Leu133 (Fig. 3).

The formation of three crucial interactions with Asn204, Glu141, and Leu133, along with other important hydrophobic interactions, like, Tyr84, Leu133, Pro134, Glu141, Phe157, Thr176, Asn204, Val205 and Ile276, indicate the formation of stable binding of the ligand with the enzyme; thus, it may have the capability to inhibit the functioning of the MTHFD2 enzyme^6^. Likewise, the molecule, UNPD224655, displayed hydrogen bond interaction with the side chain of Glu141 amino acid via 2-methoxy-phenol moiety, the side chain of Asn204 via 2,3-dihydro furan-3-yl-methanol group, and with the side chain of Ile276 via phenyl acrylaldehyde moiety of the natural product. Along with these crucial hydrogen bonds, the molecule also forms hydrophobic interaction with Leu133, Phe157, Val205, and Ile276. The acrylaldehyde and 2-methoxy phenol moieties form the hydrophobic head group as it fits inside the hydrophobic cavity of the enzyme (Fig. 3). The third molecule, UNPD230014, forms hydrogen bonding through acrylaldehyde moiety with the side chain of Ile276 residue. The rest of the interactions with amino acids Glu141 and Asn204 were formed with the –OH and –O–CH_3_ groups of the 2-methoxy phenol group. Similar to UNPD224655, here, the hydrophobic head group was formed by acrylaldehyde and 2-methoxy phenol group. Hydrophobic interactions were reported with Leu133, Phe157, Thr176, Asn204, Val205, and Val274 (Fig. 3). In the case of the natural product, UNPD224603, four hydrogen bond interactions were reported with Glu141, Asn204, Val274, and Ile276 amino acids. The –OH group of (3-methoxy phenyl) prop-2-en-1-ol form two interactions with Val274 and Ile276, and the –OH group of 2,3-dihydrofuran-3-yl-methanol moiety form an interaction with the side chain of Asn204. Also, it forms a hydrophobic head group and forms interactions with Leu133, Phe157, Asn204, Val205, and Ile276. The –OH group of 2-methoxy phenol forms hydrogen bonding with Glu141 amino acid. The fifth molecule, UNPD151821, also forms four hydrogen bonds with Gln132, Leu133, Glu141, and Asn204. The compound forms the hydrophobic interactions with Ala80, Tyr84, Leu133, Asn204, Val205, and Ile276 amino acids. The -O and -H atoms of the -OH moiety of 5-hydroxy-pyrrole-2-carbaldehyde form two hydrogen bond interactions with the side chain of Gln132 and Leu133, respectively. The remaining two interactions with the side chain of Asn204 and Glu141 were formed by -OCH_3_ and -OH moieties of 2-methoxy-phenol, respectively (Fig. 3). The sixth natural product, UNPD101160, displayed hydrogen bond interaction with the side chain of Glu141, Arg142, and Asn204 via the 2-methyl-tetrahydro furan-2-yl-methanol moiety of the compound. The remaining moiety of the compound form hydrophobic contacts with the Tyr84, Leu133, Glu141, Arg142, Phe157, Thr176, Asn204, Val205, and Ile276 amino acids. The seventh candidate, UNPD202303, forms hydrogen bonding interactions with Glu141, Arg142, and Asn204 residues. The 2,4-dimethyldec-2-en-1-ol form hydrophobic contacts with amino acids Leu133, Glu141, Phe157, and Asn204. On observing the 2D interaction plots (Fig. 3) of all seven docked candidates, common interactions with Glu141 and Asn204 amino acids were reported. Thus, from molecular docking calculations, we can predict that the shortlisted candidates showed crucial interactions with the allosteric site amino acids. Hence, they may be able to inhibit the activity of the MTHFD2 enzyme. Further, these docked complexes were subjected to molecular dynamics simulation and free energy calculations studies to check the binding stability and binding affinity within the biological environment.

Out of these seven natural compounds, ASI311667 was found to be available from Aurora building block 7. UNPD224655, i.e. (−)Balanophonin could be sourced from the tonka bean plant^69,70^. UNPD224603, i.e. (+)-Dehydrodiconiferyl alcohol can be extracted from the palm trees and is available from the A2B Chem product list^71,72^. Total synthesis of UNPD151821, i.e. Magnolamide obtained from Magnolia coco, has been reported by Ying Dong et al.^73^ and Tsz-Ying Yuen et al.^74^. The antioxidant activity of magnolamide has been discussed by Wen-Fei Chiou et al.^75^. Any specific information about the source, availability and biological effect was not obtained for compounds UNPD230014, UNPD101160, and UNPD202303 post a thorough search of the SciFinder database.

### Molecular dynamics simulations

The top docked compounds, ASI311667, UNPD224655, UNPD230014, UNPD224603, UNPD151821, UNPD101160, and UNPD202303 along with crystallographic ligand J4C and apo-protein MTHFD2 (without allosteric site binded inhibitor) was subjected to MD simulations for studying the protein dynamics and validating docking results. The MTHFD2 protein in complex with J4C, ASI311667, UNPD224655, UNPD230014, UNPD224603, UNPD151821, UNPD101160, and UNPD202303 henceforth will be referred to as MTHFD2_J4C, MTHFD2_D1, MTHFD2_D2, MTHFD2_D3, MTHFD2_D4, MTHFD2_D5, MTHFD2_D6, and MTHFD2_D7 respectively.

#### Molecular dynamics (MD) simulation of MTHFD2 and MTHFD2 docked complexes

MD simulations showing the interaction and stability of compound-protein complexes were performed to investigate the stability, dynamics, and conformational changes of the docked complexes of MTHFD2 and MTHFD2 with reference inhibitors J4C. In protein simulations, an optimal timeframe balances the observation of critical conformational changes and interactions without excessive computational burden. Striking this equilibrium prevents overlooking significant dynamics while avoiding impractical computational demands. Convergence is crucial to ensure that the system has adequately sampled the energy landscape of the conformational space and that the observed behaviours are statistically meaningful. The convergence was confirmed by the stability as observed in RMSD plots. The timescale of 300 ns also ensured to extract frames from a stable 200 ns plateau region for binding free energy calculations. In this study, we examined RMSD, RMSF, Rgyr, H-bonds, and Free energy landscape (FEL) plots for all MD simulation systems. The binding free energy of all complexes was computed for the final stable 200 ns trajectory. The mean and variance for key metrics such as RMSD, RMSF, and H-bond analyses were calculated across all frames within a single long trajectory of 300 ns. This thorough methodology provides significant statistical insights specific to each trajectory.

#### Stability of MTHFD2 protein and MTHFD2 docked complexes

**Figure 4:**
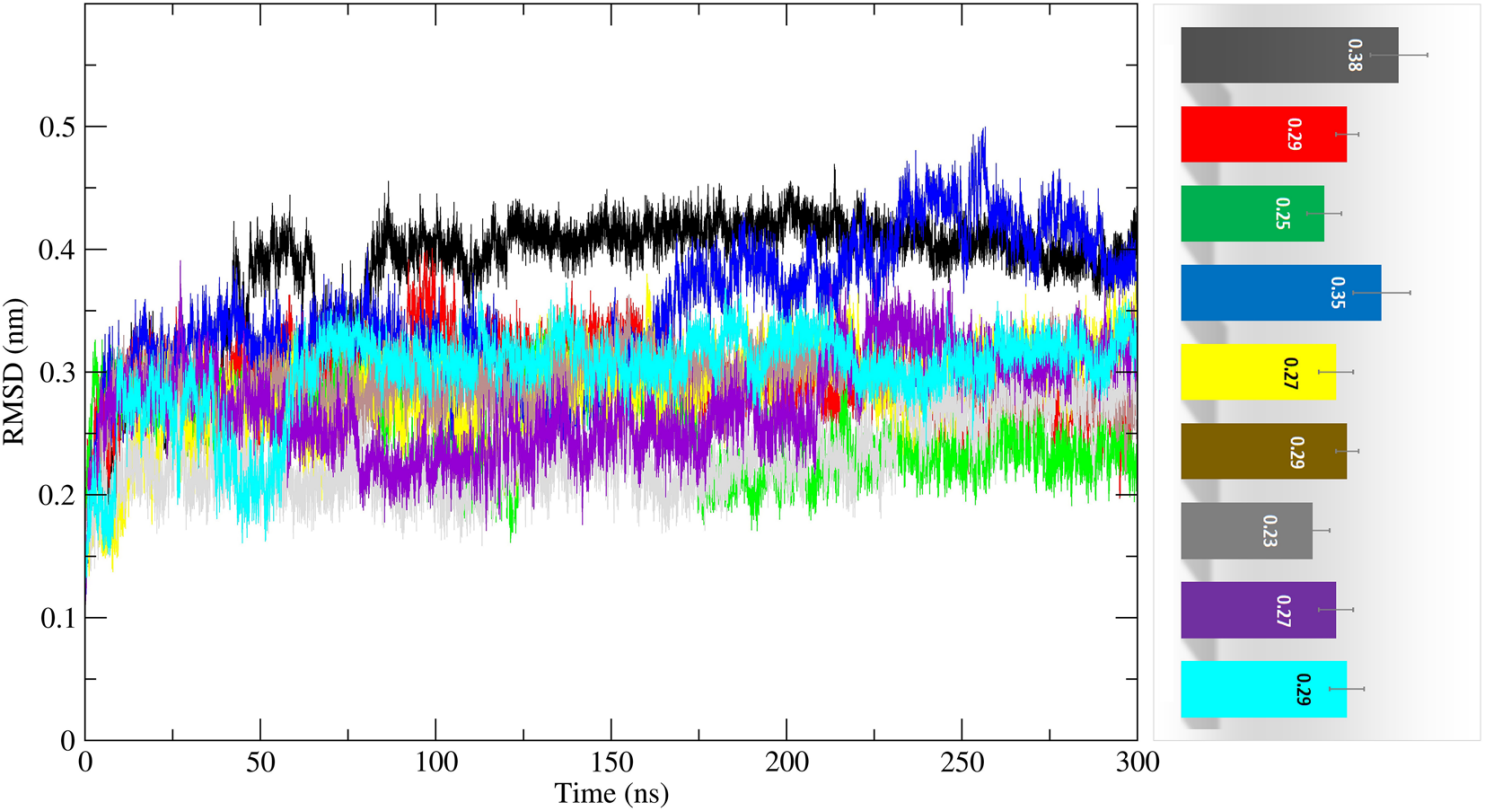
Molecular dynamics analysis of MTHFD2 and MTHFD2 in complexes with docked compounds, computing the RMSD deviation (nm) vs function of time (300 ns) of the protein Cα backbone atoms. The colour representation is MTHFD2 (black), MTHFD2_J4C (red), MTHFD2_D1 (cyan), MTHFD2_D2 (green), MTHFD2_D3 (blue), MTHFD2_D4 (yellow), MTHFD2_D5 (brown), MTHFD2_D6 (grey), and MTHFD2_D7 (purple).

The root mean square deviation (RMSD) values for the protein Cα backbone of MTHFD2 and MTHFD2 docked complexes were computed for the duration of the 300 ns. This was done in order to assess the stability and dynamics of MTHFD2 in the presence of compounds, as well as to gain molecular insights into the binding interactions of compounds. The RMSD is a measure of the structural variation in C backbones from their initial location to their final position during the course of the simulation trajectory. For the simulation system to be stable, lower RMSD values should be obtained. The RMSD graphs plotted for the docked compounds complexed with MTHFD2 protein are shown in Fig. 4.

The mean RMSD values for apo-MTHFD2, MTHFD2_J4C, MTHFD2_D1, MTHFD2_D2, MTHFD2_D3, MTHFD2_D4, MTHFD2_D5, MTHFD2_D6, and MTHFD2_D7 were 0.38 ± 0.05 nm, 0.29 ± 0.02 nm, 0.29 ± 0.03 nm, 0.25 ± 0.03 nm, 0.35 ± 0.05 nm, 0.27 ± 0.03 nm, 0.29 ± 0.02 nm, 0.23 ± 0.03 nm, and 0.27 ± 0.03 nm respectively. The RMSD value of the apo-MTHFD2 protein was comparatively higher than MTHFD2 docked complexes. All the compounds docked in MTHFD2 have RMSD values lesser or equivalent to MTHFD2 in complex with reference crystallographic ligand J4C except MTHFD2_D3. In particular, the compounds MTHFD2_D1, MTHFD2_D2, MTHFD2_D4, MTHFD2_D6, and MTHFD2_D7 showed lesser RMSD values compared to the reference ligand. This implies that during the calculations, the compounds MTHFD2_D1, MTHFD2_D2, MTHFD2_D4, MTHFD2_D6, and MTHFD2_D7 persisted to be stable in complex with MTHFD2 protein.

**Figure 5:**
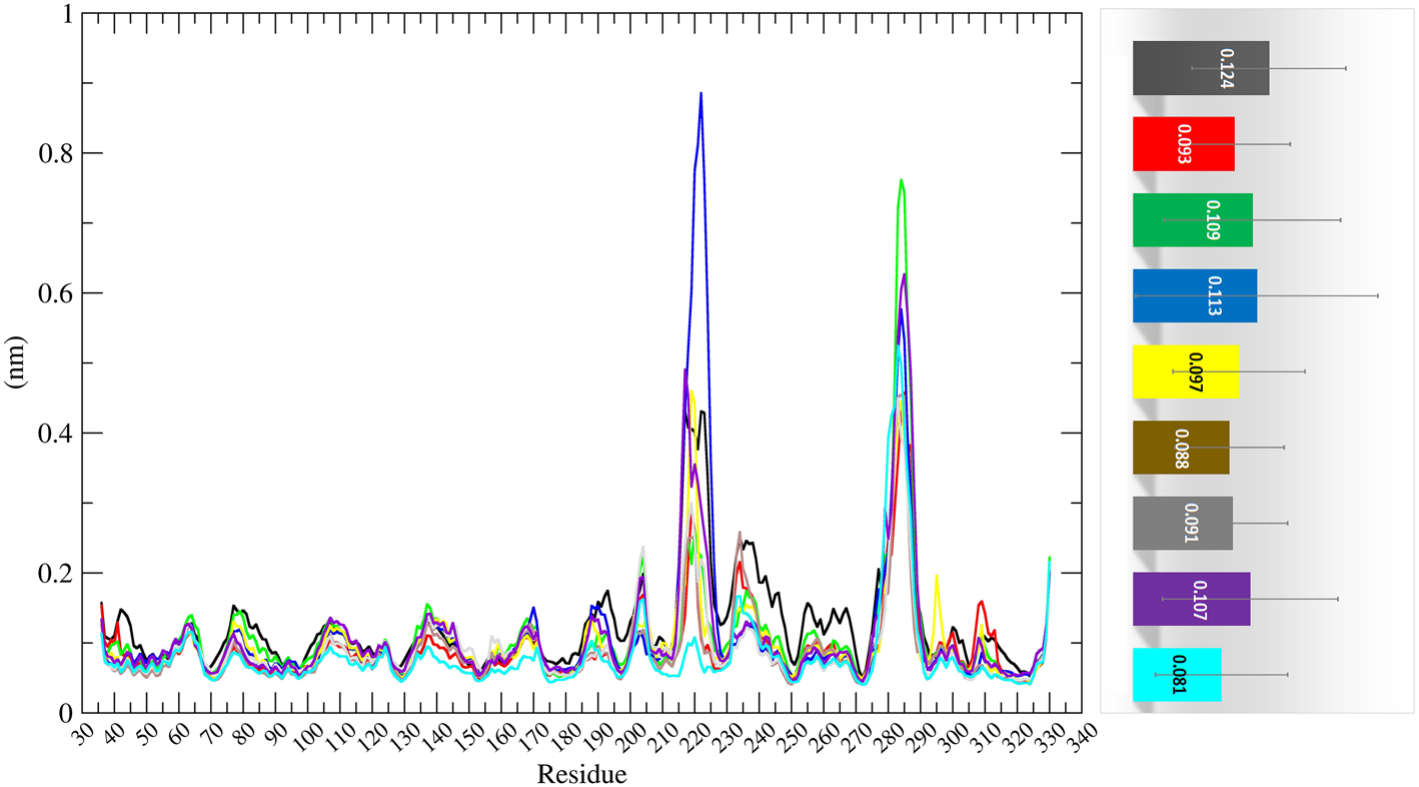
Molecular dynamics analysis of MTHFD2 and MTHFD2 in complexes with docked compounds, computing the RMSF deviation (nm) vs function of time (300 ns) of the protein Cα backbone atoms for each residue. The colour representation is MTHFD2 (black), MTHFD2_J4C (red), MTHFD2_D1 (cyan), MTHFD2_D2 (green), MTHFD2_D3 (blue), MTHFD2_D4 (yellow), MTHFD2_D5 (brown), MTHFD2_D6 (grey), and MTHFD2_D7 (purple).

**Figure 6:**
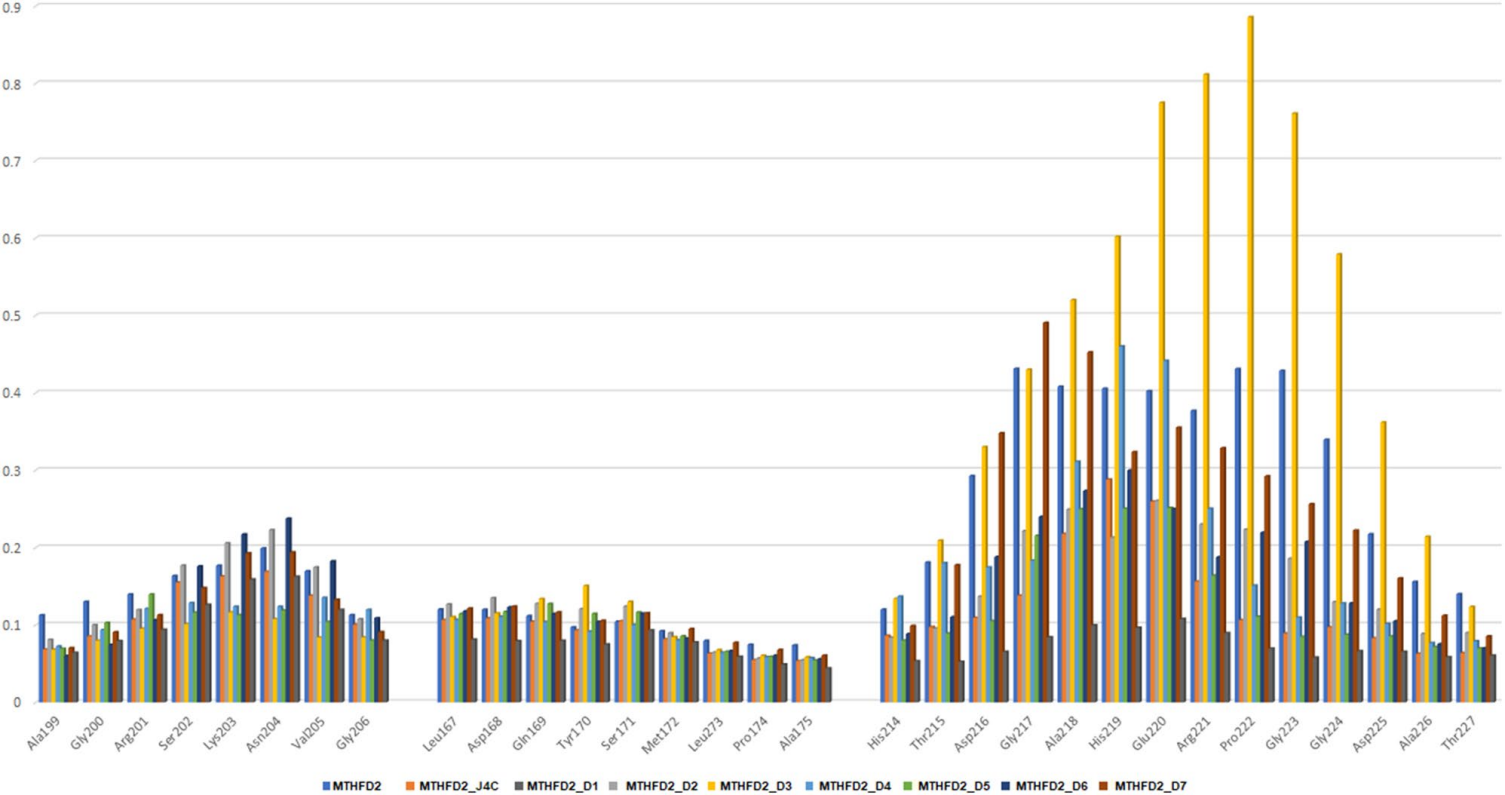
The bar plot depicting the RMSF values (nm) of the protein Cα backbone atoms computed for 300 ns for three flexible loops βe-αE, αD2ʹ-αD3ʹ and αEʹ-βfʹ.

The root mean squared fluctuations (RMSF) plots were generated for 300 ns to analyse the residue-wise fluctuations. For comparison of flexible residues of protein with respect to docked complexes, the RMSF values for various docked compounds were overlaid on the apo-MTHFD2 (Fig. 5). The mean RMSF values for MTHFD2, MTHFD2_J4C, MTHFD2_D1, MTHFD2_D2, MTHFD2_D3, MTHFD2_D4, MTHFD2_D5, MTHFD2_D6, and MTHFD2_D7 were 0.124 ± 0.07 nm, 0.093 ± 0.05 nm, 0.081 ± 0.06 nm, 0.109 ± 0.08 nm, 0.113 ± 0.11 nm, 0.097 ± 0.06 nm, 0.088 ± 0.05 nm, 0.091 ± 0.05 nm, and 0.107 ± 0.08 nm respectively. Similar to RMSD, the RMSF values for the apo-MTHFD2 are higher when compared to all the docked complexes, indicating lesser residue level fluctuations in the docked complex. Previous literature and crystal structures of MTHFD2 have revealed the conformational changes in three loops βe–αE (199–206), αD2ʹ–αD3ʹ (167–175), and αEʹ–βfʹ (214–227) of protein on binding with the inhibitors at the allosteric site^6,76^. It was evident from the RMSF analysis, as shown in Fig. 5 that the αEʹ–βfʹ loop (residue 214–227) was the most flexible with the highest deviations. A similar trend was also observed for the region with residues 275–290. Over the entire course of 300 ns simulations, we compared the RMSF values of the three loops for MTHFD2 docked complexes with the reference ligand J4C and apo-protein. The RMSF computed allowed us to identify the residues among the three loops of MTHFD2 that displayed the highest fluctuations during the simulations (Fig. 6).

**Figure 7:**
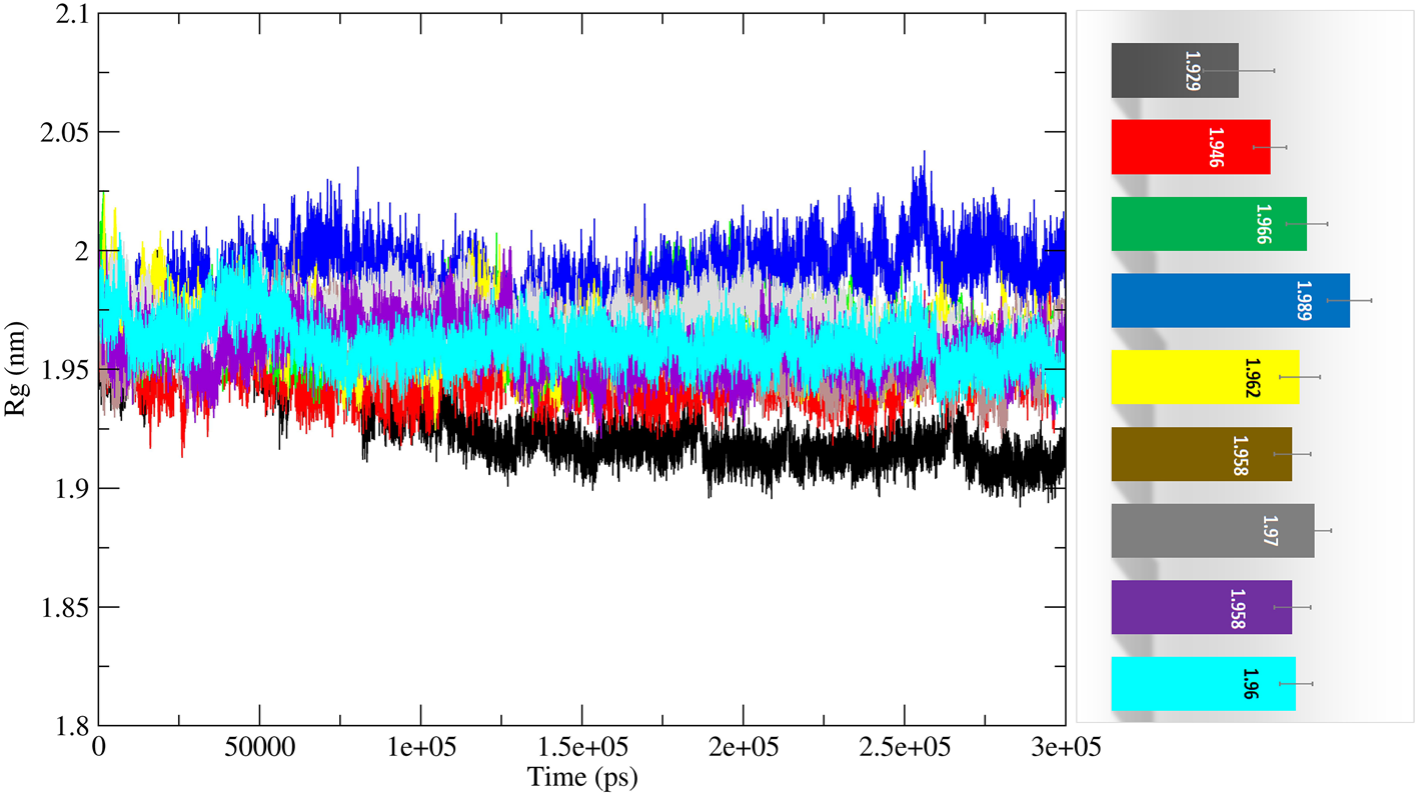
Molecular dynamics analysis of MTHFD2 and MTHFD2 in complexes with docked compounds, computing the RoG deviation (nm) vs function of time (300 ns) of the protein Cα backbone atoms for each residue. The colour representation is MTHFD2 (black), MTHFD2_J4C (red), MTHFD2_D1 (cyan), MTHFD2_D2 (green), MTHFD2_D3 (blue), MTHFD2_D4 (yellow), MTHFD2_D5 (brown), MTHFD2_D6 (grey), and MTHFD2_D7 (purple).

The deviation of most residues in the αD2ʹ–αD3ʹ loop (167–175) was almost equivalent for apo-MTHFD2 protein and MTHFD2 with the allosterically docked compounds. The βe–αE loop (199–206) showed higher deviation for apo-MTHFD2 protein in comparison to the docked complexes of MTHFD2 protein. The third αEʹ–βfʹ loop (214–227) display the highest deviation in the apo-MTHFD2 protein compared to the docked complexes of MTHFD2 except for the MTHFD2_D3 compound. The MTHFD2_D3 showed the highest deviation amongst all the residues of the loop. The representative structures from all the trajectories were also superposed to compare and visualize the displacement of all three loops in the presence of docked complexes at the allosteric site (Supplementary Fig. S4). As observed in the earlier reports, a similar conformational change in αEʹ–βfʹ loop was observed in the average structures of all the simulations, leading to its displacement away from the allosteric site of MTHFD2^76^. Overall, the RMSD and RMSF analyses revealed that the docked complexes at the allosteric site exhibited similar or lower fluctuations, thus, elucidating lower variability at the residue level. Furthermore, all 300 ns simulations were submitted to the Radius of Gyration (RoG) calculations. The RoG compares the shape of the protein at each trajectory to the experimentally attainable hydrodynamic radius. Figure 7 depicts the average RoG values of protein C backbones for all compounds and the reference ligand with MTHFD2.

The mean RoG values for MTHFD2, MTHFD2_J4C, MTHFD2_D1, MTHFD2_D2, MTHFD2_D3, MTHFD2_D4, MTHFD2_D5, MTHFD2_D6, and MTHFD2_D7 were 1.929 ± 0.019 nm, 1.946 ± 0.009 nm, 1.960 ± 0.009 nm, 1.966 ± 0.011 nm, 1.989 ± 0.012 nm, 1.962 ± 0.011 nm, 1.958 ± 0.010 nm, 1.970 ± 0.009 nm, and 1.958 ± 0.010 nm respectively. The RoG values of all the docked systems were similar, thus, demonstrating that the overall structure of the docked complexes with MTHFD2 protein is consistent. However, compared to the apo-MTHFD2, all docked complexes exhibited marginally higher RoG values, indicating a minor increase in the protein hydrodynamic radius in the presence of the docked compounds (Fig. 7). We also calculated the surface accessible solvent area (SASA) component over the full trajectory for all systems. The SASA calculations access the surface area of protein that is accessible to the solvent. The SASA values were significantly similar to the apo-MTHFD2, MTHFD2_J4C, and MTHFD2 docked complexes (data not shown). Overall, the RMSD, RMSF, Rgyr, and SASA analyses revealed that the docked compounds displayed higher stability and stable dynamic pattern when compared to the apo-MTHFD2 protein and MTHFD2 complexed experimentally known inhibitor/ligand (J4C).

#### Molecular interactions of MTHFD2 with identified compounds from docking

**Figure 8:**
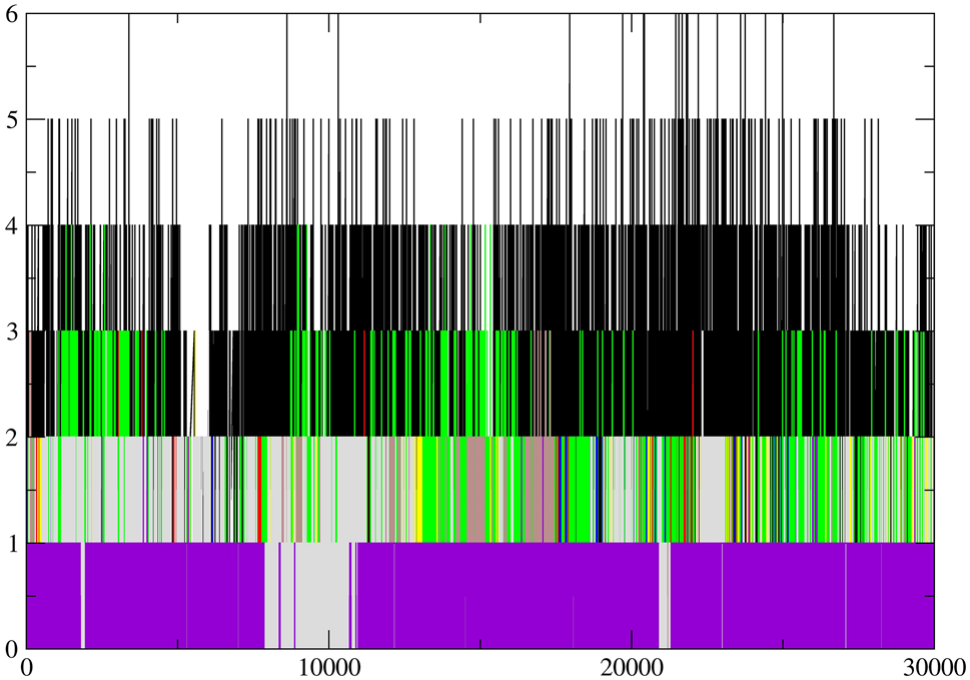
Computation of H-bond formation between MTHFD2 and docked compounds for 300 ns duration. The colour representation is MTHFD2_J4C (black), MTHFD2_D1 (purple), MTHFD2_D2 (red), MTHFD2_D3 (green), MTHFD2_D4 (blue), MTHFD2_D5 (yellow), MTHFD2_D6 (brown), and MTHFD2_D7 (grey).

**Figure 9:**
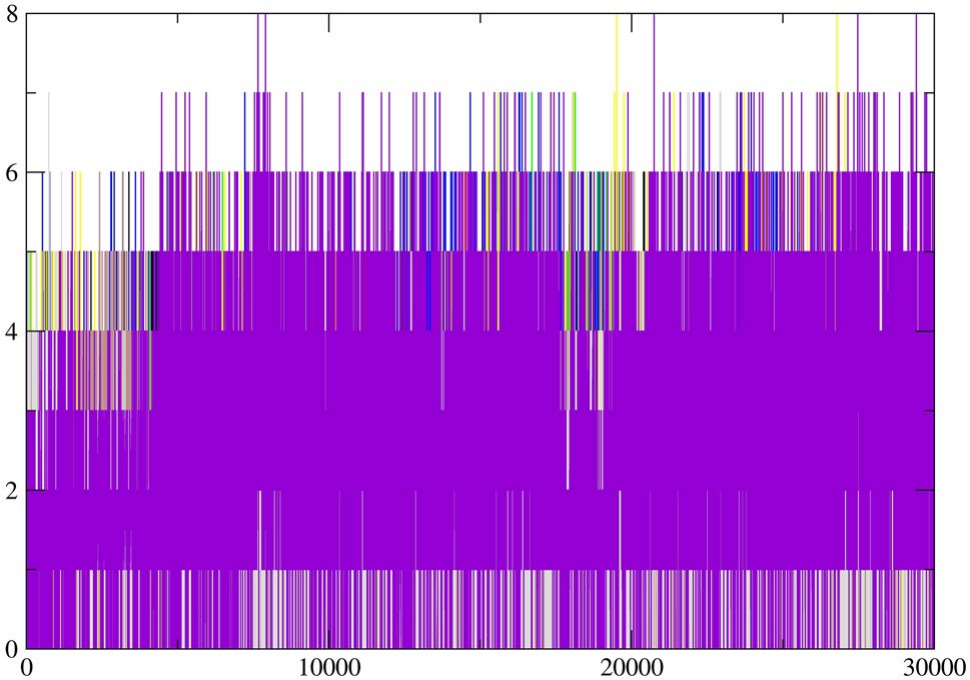
Computation of H-bonds formation between MTHFD2 and reference ligand J4C in all docked complexes for 300 ns. The colour representation is MTHFD2_J4C (black), MTHFD2_D1 (purple), MTHFD2_D2 (red), MTHFD2_D3 (green), MTHFD2_D4 (blue), MTHFD2_D5 (yellow), MTHFD2_D6 (brown), and MTHFD2_D7 (grey).

H-bond formation indicates specificity and molecular interactions between the protein and inhibitors in the complexes. Figure 8 depicts the calculated mean values for H-bonds formed between MTHFD2 protein and docked compounds. The average H-bond values for MTHFD2_J4C, MTHFD2_D1, MTHFD2_D2, MTHFD2_D3, MTHFD2_D4, MTHFD2_D5, MTHFD2_D6, and MTHFD2_D7, were 1.687 ± 1.10, 0.763 ± 0.42, 0.297 ± 0.486, 0.686 ± 0.73, 0.091 ± 0.29, 0.013 ± 0.35, 0.258 ± 0.46, and 0.599 ± 0.56 respectively. We also have computed the average H-bond values for the J49 ligand (active site ligand) bound in all the trajectories of MTHFD2 in complex with seven docked compounds to assess the interaction of the J49 with the protein with respect to the presence of the compounds at the allosteric site. The average H-bond values for J49 in different complexes MTHFD2_J4C, MTHFD2_D1, MTHFD2_D2, MTHFD2_D3, MTHFD2_D4, MTHFD2_D5, MTHFD2_D6, and MTHFD2_D7 were 1.687 ± 1.10, 2.586 ± 1.38, 1.633 ± 1.07, 1.429 ± 1.06, 2.014 ± 1.26, 1.784 ± 1.23, 1.361 ± 1.10, and 1.792 ± 1.21 respectively (Fig. 9). The average number of H-bonds with different inhibitors was lesser than the crystallographic reference ligand. On the other hand, the average no. of H-bonds of J49 (active site ligand) in MTHFD2_D1, MTHFD2_D4, MTHFD2_D5, and MTHFD2_D7 inhibitor complexes were higher than the complex with only J4C ligand in MTHFD2. The intra-H-bonds in MTHFD2_J4C and docked-MTHFD2 protein were also computed. The average intra-H-bonds for MTHFD2_J4C was 64.60 ± 14.10, while for the respective inhibitors, the average H-bonds ranged from 58 to 68 (data not shown).

### Binding free energy estimation and energy decomposition of MTHFD2 and MTHFD2 docked complexes with inhibitors

**Figure 10:**
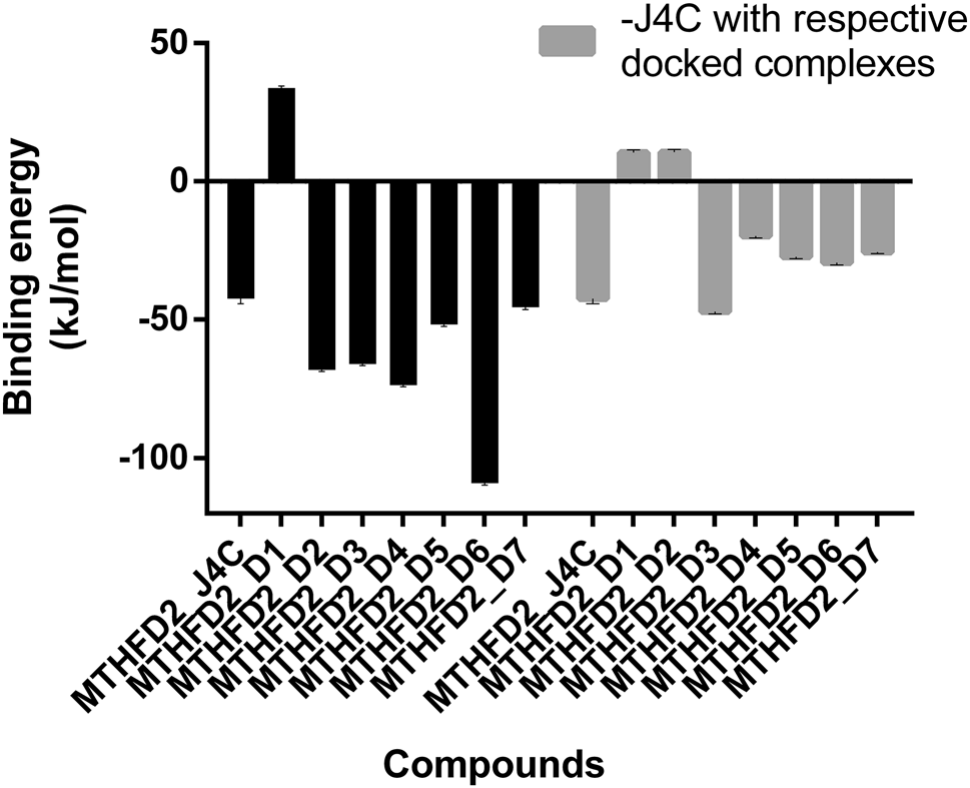
MM-PBSA Calculation of binding free energy. The total binding free energy for all the MTHFD2 docked complexes was calculated for the last 200 ns stable trajectory for 1000 frames. The binding free energy for docked complexes is black, while the binding free energy for reference ligand J4C is grey in each docked complex.

The MD trajectories of MTHFD2_J4C, MTHFD2_D1, MTHFD2_D2, MTHFD2_D3, MTHFD2_D4, MTHFD2_D5, MTHFD2_D6, and MTHFD2_D7 were used for computing binding free energies. The MM/PBSA method is commonly used to estimate the binding free energies, which estimate the non-bonded interaction energies. Using the MM-PBSA method, the binding free energy (ΔG) between the MTHFD2 protein and the selected docked compounds was calculated for the last 200 ns of stable trajectories. For accurate binding free energy calculations, thorough conformational sampling should be done, but the MM/PBSA relies on a single trajectory that might lead to incomplete energy landscape exploration. Therefore, according to the stable convergence of the simulation outcome, we extracted the last trajectories from the last 200 ns. From the MD simulations outcome, 1000 frames from the last 200 ns of trajectories were considered for the computation. The estimated values of ΔG (kJ mol^−1^) calculated for all MD simulations involving MTHFD2_J4C and docked MTHFD2 complexes are shown in Fig. 10. All the compounds showed better binding free energy values than the MTHFD2_J4C except the MTHFD2_D1. The best one was found to be MTHFD2_D6, followed by MTHFD2_D4, MTHFD2_D2, MTHFD2_D3, MTHFD2_D5, and MTHFD2_D7. We also computed the binding energy for J49 (active site inhibitor) with protein MTHFD2 in the presence of the docked inhibitors (Fig. 10).

In most docked complexes, the binding energy for J49 decreases except for MTHFD2_D3, which was slightly equivalent to the only MTHFD2_J4C complex. The decrease in the reference ligand J4C binding energy in the presence of the docked inhibitors suggests the role of inhibitors in the allosteric site. The identified inhibitors may interfere with and decrease the binding affinity of the J4C to the MTHFD2. The experimental results may identify more information on the effect of docked compounds.

#### PCA and FEL analysis of MTHFD2 and MTHFD2 docked complexes

**Figure 11:**
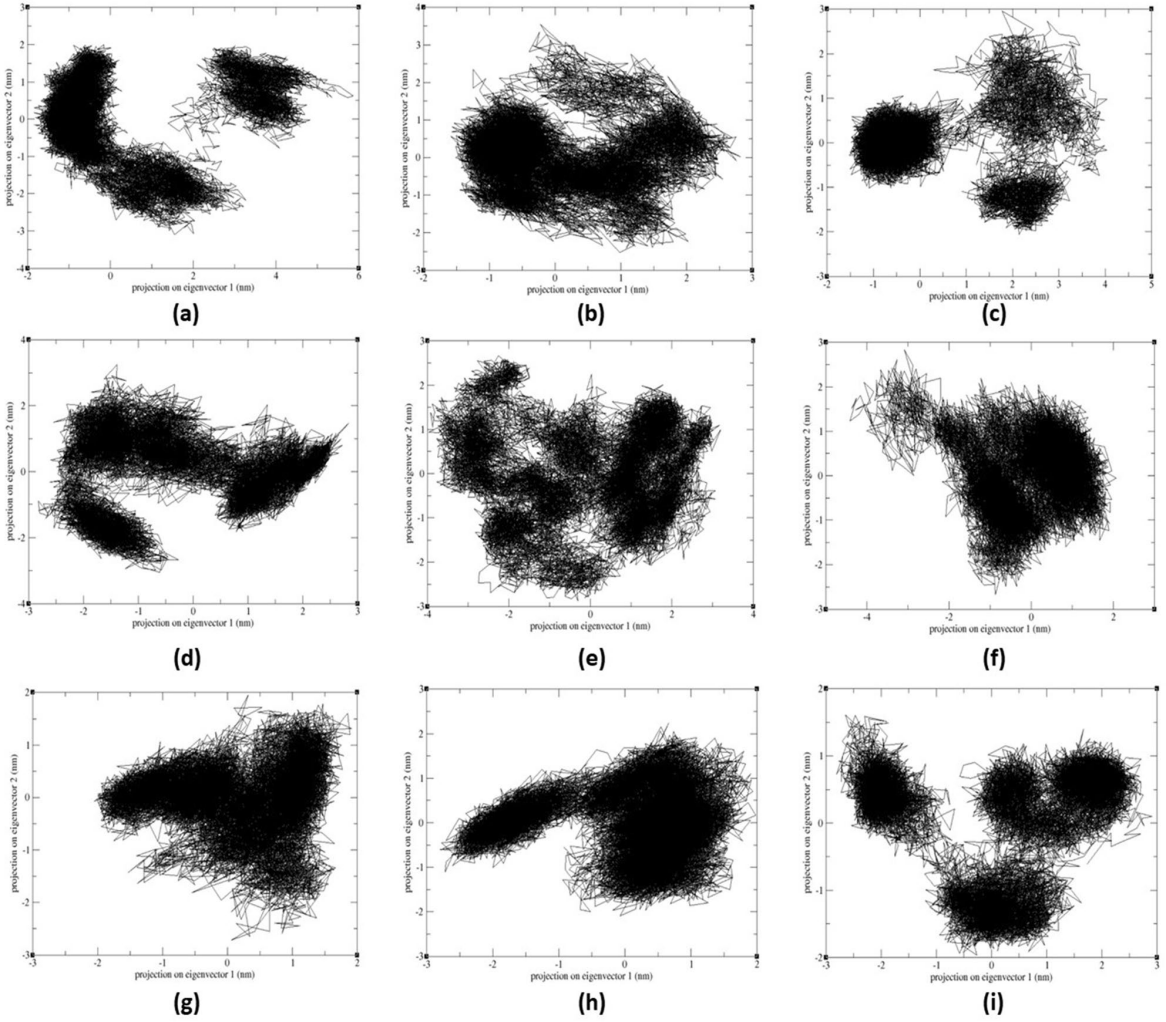
Principle component analysis (PCA) of MTHFD2 and MTHFD2 docked complexes for 300 ns showing 2D scatter plot projecting the motion of the protein in phase space for the two principal components, PC1 and PC3. The panel is represented as MTHFD2 (**a**), MTHFD2_J4C (**b**), MTHFD2_D1 (**c**), MTHFD2_D2 (**d**), MTHFD2_D3 (**e**), MTHFD2_D4 (**f**), MTHFD2_D5 (**g**), MTHFD2_D6 (**h**), and MTHFD2_D7.

**Figure 12:**
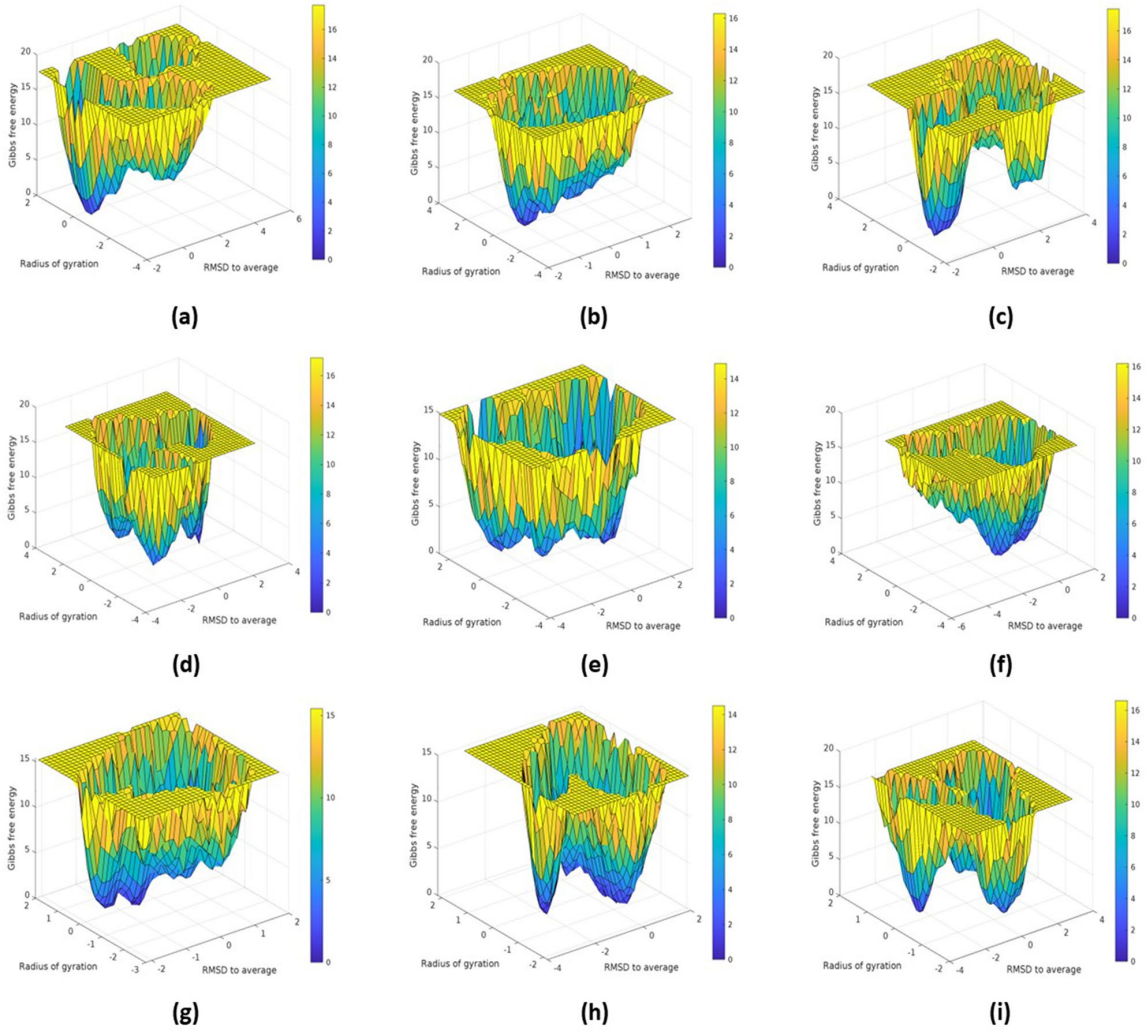
Free energy landscape (FEL) of MTHFD2 and MTHFD2 docked complexes for 300 ns MDS. The panel is represented as MTHFD2 (**a**), MTHFD2_J4C (**b**), MTHFD2_D1 (**c**), MTHFD2_D2 (**d**), MTHFD2_D3 (**e**), MTHFD2_D4 (**f**), MTHFD2_D5 (**g**), MTHFD2_D6 (**h**), and MTHFD2_D7 (**i**).

Principal Component Analysis (PCA) examined the conformational space and transitions in the apo and docked complex structures. The PCA reduces the complexity of the simulated trajectories by isolating C atom collective motion while retaining the majority of the variation. It computes the covariance matrix of positional fluctuations for backbone atoms, which is used to understand the dynamics and coherent motions of MTHFD2 in the absence/presence of compounds. Figure 11 depicts the trajectory of two primary principal components, PC1 and PC2, for apo-MTHFD2, MTHFD2_J4C, and MTHFD2 docked complexes. The FEL is plotted to better comprehend the protein's conformational change during the simulation. The FEL 3D graphs were plotted against two primary components, i.e., RMSD and Rgyr (Fig. 12). Each protein–ligand combination had a unique FEL pattern. Based on the energy, the dark blue color spots represent minimal energy and highly favoured protein conformations, whereas the yellow color spots represent undesirable conformations (Fig. 12).

The system MTHFD2_J4C, MTHFD2_D3, MTHFD2_D4, and MTHFD2_D5 had a very similar pattern of FEL with one broad peak in the funnel. On the other hand, apo-MTHFD2, MTHFD2_D1, MTHFD2_D3, MTHFD2_D6, and MTHFD2_D7 had multiple peaks in the funnel. The PCA and FEL analysis profoundly concluded that the MTHFD2 had a very distinct behaviour with different ligands and multi-conformational structures found in the entire simulations. This ascertains that the docked protein-ligands compounds had a role in displaying stable protein conformational structures and overall dynamics. The identified compounds must play a role in protein distinct patterns and dynamics when compared to the apo-protein.

### Conclusions

The MTHFD2 enzyme is identified as one of the new and attractive anticancer drug targets. As per recent studies, MTHFD2 has an allosteric binding site that coexists with the substrate analogue. It is reported that binding of the inhibitors in the allosteric site leads to disruption in the enzyme mechanism. Also, allosteric site inhibition is gaining importance owing to the increase in the selectivity of the inhibitors and reduction in the emergence of drug resistance. Given this importance, our work presents the sheer use of various computational techniques for identifying and validating hit molecules. The reported crystallized complexes of allosterically bound MTHFD2 enzymes were selected. The selected protein complexes were superimposed to generate a single coordinate file which was further subjected to pharmacophore modeling. Validation of pharmacophores was performed to check the specificity of the models. To conduct the virtual screening (VS) process, a dataset of 2,36,561 molecules of various natural product (NP) databases was selected and prepared. After screening, we retrieved 494 unique candidates, which were further subjected to molecular docking and HYDE assessment. The shortlisted candidates were prepared as per Lipinski’s rule of five and Veber's rule. From docking and HYDE assessment, we obtained 72 molecules, which were further shortlisted to 63 based on the drug-likeness studies. On conducting ADMET studies, only 20 molecules were selected. Out of 20, only seven were selected based on the presence of interaction with important amino acids, i.e., glutamic acid (Glu141) and asparagine (Asn204). These seven candidates were subjected to molecular dynamics simulations. In simulations, we examined RMSD, RMSF, Rgyr, and H-bonds plots for all the docked systems, along with reference and apo-protein. From the outcome of RMSD and RMSF analysis, we observed that all the docked complexes displayed stable behaviour in comparison to the selected reference and apo-protein. Also, from protein RMSF analysis, we studied the protein conformational changes in three loops βe–αE (199–206), αD2ʹ–αD3ʹ (167–175), and αEʹ–βfʹ (214–227) on binding with the inhibitors at the allosteric site. The deviation of the residues in the range of 167–175 was almost equivalent for apo-MTHFD2 protein and docked MTHFD2 complexes. The amino acids from 199 to 206 showed lesser deviation in docked complexes when compared with the apo-MTHFD2 protein. The third αEʹ–βfʹ loop (214–227) display the highest deviation in the apo-MTHFD2 protein than the docked complexes of MTHFD2 except for the MTHFD2_D3 compound. Also, as per the earlier reports, a similar conformational change in the αEʹ–βfʹ loop was observed in the average superimposed structures of all the simulations, leading to its displacement away from the allosteric site of MTHFD2. From RoG analysis, marginal higher fluctuations were observed in docked complexes representing the increase in the protein hydrodynamic radius in the presence of the docked compounds. Hydrogen bond analysis was performed to check the average interactions formed in the docked complexes (allosteric site) and J49 (active site inhibitor) and intra-H-bonding. The average number of H bonding was different in all the docked complexes, and the average number of intra-H-bonds was 58–68 displaying the presence of bonding during the simulations. From binding free energy calculations, we observed that all the complexes except (MTHFD2_D1) showed higher binding energy. Also, in the case of calculation of the binding energy of J49 (active site inhibitor), it was observed that the presence of docked complexes causes a decrease in the energy suggesting the role of inhibitors in the allosteric site of the protein. The PCA and FEL analysis revealed that the MTHFD2 enzyme had a distinct behaviour with different ligands and multi-conformational structures found in the simulations. Also, the docked complexes play an essential role in protein conformational change and overall dynamics, a behaviour of stable protein–ligand complexes. Overall, based on the outcome of the results in the current study, these complexes can contribute to the development of potential drug-like natural products that have the capability of inhibiting the MTHFD2 enzyme. The synthesis of these compounds is under process, and after synthesis, they will be evaluated for their anticancer activities.

### Supplementary Information

Supplementary Information.

## Data Availability

The datasets used and/or analysed in the current study are available from the corresponding author upon reasonable request.
