# Supplementary material for: Targeting allosteric binding site in methylenetetrahydrofolate dehydrogenase 2 (MTHFD2) to identify natural product inhibitors via structure-based computational approach
Source: Sci Rep. 2023 Oct 23;13:18090. doi: 10.1038/s41598-023-45175-3 (PMC10593809; doi:10.1038/s41598-023-45175-3)
Supplement: Supplementary file 1 — Supplementary Information. [file 41598_2023_45175_MOESM1_ESM.docx]

**Targeting Allosteric binding site in Methylenetetrahydrofolate Dehydrogenase 2 (MTHFD2) to identify natural product inhibitors via Structure-based Computational Approach**

Nisarg Rana^a^, Dhaval Patel^b^, Meet Parmar^b^, Nandini Mukherjee^a^, Prakash C Jha^c^, Anu Manhas^a*^

^a^Department of Chemistry, School of Energy Technology, Pandit Deendayal Energy University-382426

^b^Department of Industrial Biotechnology, Gujarat Biotechnology University, Gandhinagar, India

^c^School of Applied Material Sciences, Central University of Gujarat, Gandhinagar-382030

Email: [anu.manhas15@gmail.com](mailto:anu.manhas15@gmail.com), [Anu.Manhas@sot.pdpu.ac.in](mailto:Anu.Manhas@sot.pdpu.ac.in), +91-9149873239

**Supplementary table 1:** List of the generated common feature pharmacophore models and their inter-feature distance (geometric constraint).

| **Model Number** | **Pharmacophore** | **Features** | **Interfeature-distance** |
| --- | --- | --- | --- |
| 1 | Y(1.11) Z(2.11) Z(3.11) A(4.11) A(5.11)  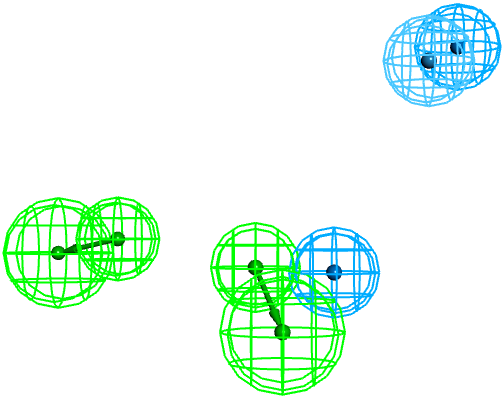 | Z(2.11) 🡪 Z(3.11) | 10.493 |
|  |  | Y(1.11) 🡪 Z(2.11) | 08.694 |
|  |  | Y(1.11) 🡪 Z(3.11) | 03.418 |
|  |  | A(4.11) 🡪 Z(2.11) | 03.032 |
|  |  | A(4.11) 🡪 Y(1.11) | 10.153 |
|  |  | A(4.11) 🡪 Z(3.11) | 11.954 |
|  |  | A(4.11) 🡪 A(5.11) | 06.732 |
|  |  | A(5.11) 🡪 Z (2.11) | 09.631 |
|  |  | A(5.11) 🡪 Y(1.11) | 14.567 |
|  |  | A(5.11) 🡪 Z(3.11) | 15.075 |
|  |  |  |  |
| 2 | R(1.11) Z(2.11) Z(3.11) A(4.11) A(5.11)  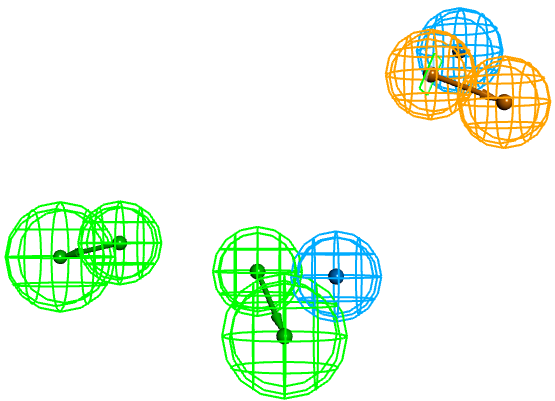 | R(1.11) 🡪 Z(2.11) | 08.425 |
|  |  | R(1.11) 🡪 Z(3.11) | 03.044 |
|  |  | Z (2.11) 🡪 Z(3.11) | 10.493 |
|  |  | A(4.11) 🡪 Z(2.11) | 03.032 |
|  |  | A(4.11) 🡪 Z(3.11) | 11.954 |
|  |  | A(4.11) 🡪 R(1.11) | 09.930 |
|  |  | A(5.11) 🡪 A(4.11) | 06.732 |
|  |  | A(5.11) 🡪 Z (2.11) | 09.631 |
|  |  | A(5.11) 🡪 Z(3.11) | 15.705 |
|  |  | A(5.11) 🡪 R(1.11) | 14.327 |
|  |  |  |  |
| 3 | R(1.11) Z(2.11) Z(3.11) A(4.11) A(5.11)  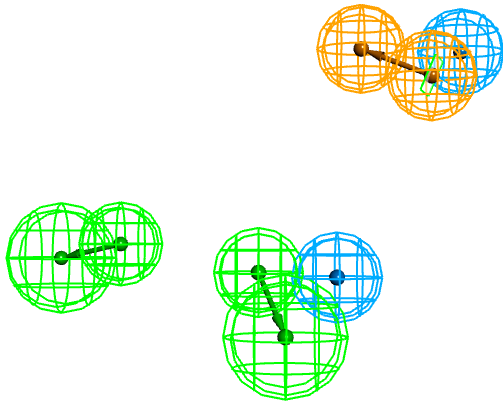 | R(1.11) 🡪 Z(2.11) | 08.425 |
|  |  | R(1.11) 🡪 Z(3.11) | 03.044 |
|  |  | Z(2.11) 🡪 Z(3.11) | 10.493 |
|  |  | A(4.11) 🡪 Z(2.11) | 03.032 |
|  |  | A(4.11) 🡪 Z(3.11) | 11.954 |
|  |  | A(4.11) 🡪 R(1.11) | 09.930 |
|  |  | A(5.11) 🡪 A(4.11) | 06.732 |
|  |  | A(5.11) 🡪 Z(2.11) | 09.631 |
|  |  | A(5.11) 🡪 Z(3.11) | 15.705 |
|  |  | A(5.11) 🡪 R(1.11) | 14.327 |
|  |  |  |  |

| **Continued Supplementary table 1…….** | | | |
| --- | --- | --- | --- |
| 4 | Y(1.11) Z(2.11) Z(3.11) H(4.11) D(5.11)  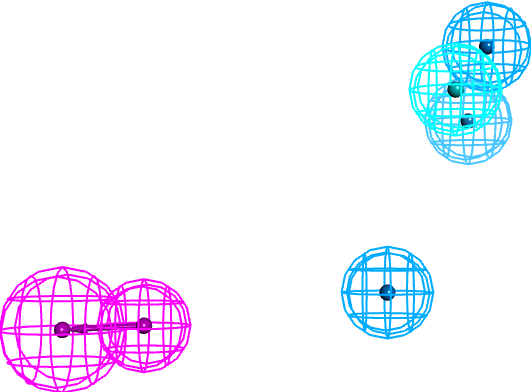 | Y(1.11) 🡪 Z(2.11) | 07.904 |
|  |  | Y(1.11) 🡪 Z(3.11) | 03.054 |
|  |  | H(4.11) 🡪 Y(1.11) | 03.359 |
|  |  | Z(2.11) 🡪 Z(3.11) | 10.211 |
|  |  | H(4.11) 🡪 Z(2.11) | 07.889 |
|  |  | H(4.11) 🡪 Z(3.11) | 02.964 |
|  |  | D(5.11) 🡪 Z(2.11) | 09.042 |
|  |  | D(5.11) 🡪 Z(3.11) | 16.725 |
|  |  | D(5.11) 🡪 Y(1.11) | 14.950 |
|  |  | D(5.11) 🡪 H(4.11) | 14.337 |
|  |  |  |  |
| 5 | R(1.11) Z(2.11) Z(3.11) H(4.11) D(5.11)  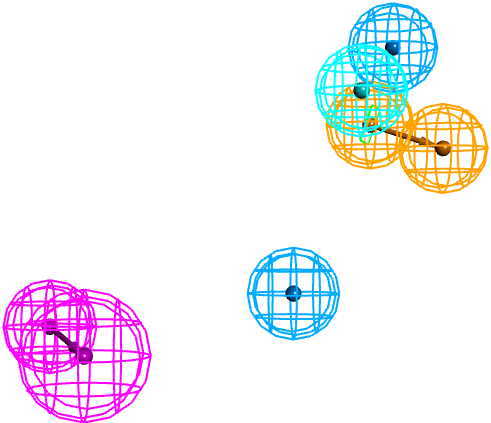 | R(1.11) 🡪 H(4.11) | 03.038 |
|  |  | R(1.11) 🡪 Z(2.11) | 07.576 |
|  |  | R(1.11) 🡪 Z(3.11) | 03.110 |
|  |  | H(4.11) 🡪 Z(2.11) | 07.889 |
|  |  | H(4.11) 🡪 Z(3.11) | 02.964 |
|  |  | Z(2.11) 🡪 Z(3.11) | 10.211 |
|  |  | D(5.11) 🡪 Z(2.11) | 09.042 |
|  |  | D(5.11) 🡪 Z(3.11) | 16.725 |
|  |  | D(5.11) 🡪 R(1.11) | 14.626 |
|  |  | D(5.11) 🡪 H(4.11) | 14.337 |
|  |  |  |  |
| 6 | R(1.11) Z(2.11) Z(3.11) H(4.11) D(5.11)  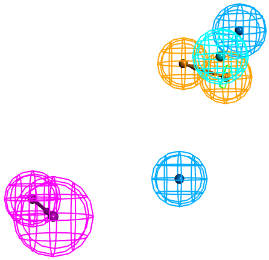 | R(1.11) 🡪 H(4.11) | 03.038 |
|  |  | R(1.11) 🡪 Z(2.11) | 07.576 |
|  |  | R(1.11) 🡪 Z(3.11) | 03.110 |
|  |  | H(4.11) 🡪 Z(2.11) | 07.889 |
|  |  | H(4.11) 🡪 Z(3.11) | 02.964 |
|  |  | Z(2.11) 🡪 Z(3.11) | 10.211 |
|  |  | D(5.11) 🡪 Z(2.11) | 09.042 |
|  |  | D(5.11) 🡪 Z(3.11) | 16.725 |
|  |  | D(5.11) 🡪 R(1.11) | 14.626 |
|  |  | D(5.11) 🡪 H(4.11) | 14.337 |
|  |  |  |  |
| 7 | Z(1.11) Z(2.11) H(3.11) H(4.11) A(5.11)  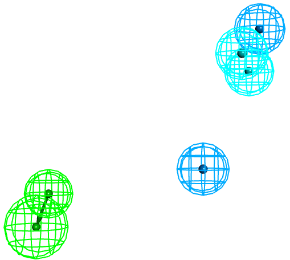 | Z(1.11) 🡪 Z(2.11) | 10.211 |
|  |  | Z(1.11) 🡪 H(3.11) | 07.904 |
|  |  | Z(1.11) 🡪 H(4.11) | 07.889 |
|  |  | Z(2.11) 🡪 H(3.11) | 03.054 |
|  |  | Z(2.11) 🡪 H(4.11) | 02.964 |
|  |  | H(3.11) 🡪 H(4.11) | 03.359 |
|  |  | A(5.11) 🡪 Z(1.11) | 11.337 |
|  |  | A(5.11) 🡪 Z(2.11) | 18.427 |
|  |  | A(5.11) 🡪 H(3.11) | 16.331 |
|  |  | A(5.11) 🡪 H(4.11) | 16.555 |
|  |  |  |  |
| 8 | Y(1.11) Z(2.11) Z(3.11) H(4.11) A(5.11)  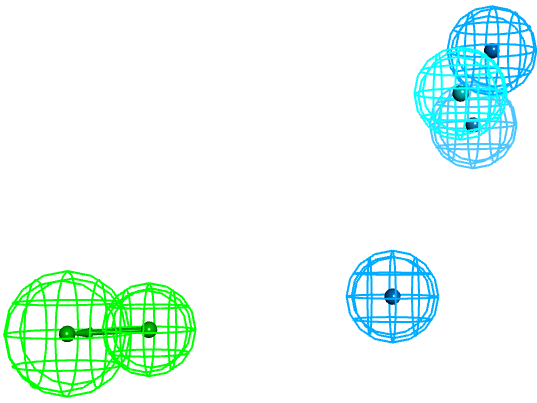 | Y(1.11) 🡪 Z(2.11) | 07.904 |
|  |  | N(1.11) 🡪 Z(3.11) | 03.054 |
|  |  | Y(1.11) 🡪 H(4.11) | 03.359 |
|  |  | H(4.11) 🡪 Z(2.11) | 07.889 |
|  |  | H(4.11) 🡪 Z(3.11) | 02.964 |
|  |  | Z(2.11) 🡪 Z(3.11) | 10.211 |
|  |  | A(5.11) 🡪 Y(1.11) | 14.950 |
|  |  | A(5.11) 🡪 Z(2.11) | 09.042 |
|  |  | A(5.11) 🡪 Z(3.11) | 16.725 |
|  |  | A(5.11) 🡪 H(4.11) | 14.337 |

| **Continued Supplementary table 1…….** | | | |
| --- | --- | --- | --- |
| 9 | Y(1.11) Z(2.11) Z(3.11) H(4.11) A(5.11)  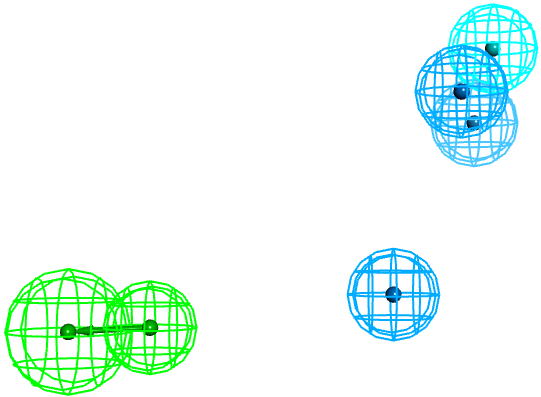 | Y(1.11) 🡪 Z(2.11) | 07.904 |
|  |  | Y(1.11) 🡪Z(3.11) | 03.359 |
|  |  | Y(1.11) 🡪 H(4.11) | 03.054 |
|  |  | H(4.11) 🡪 Z(2.11) | 10.211 |
|  |  | H(4.11) 🡪 Z(3.11) | 02.964 |
|  |  | Z(2.11) 🡪 Z(3.11) | 07.889 |
|  |  | A(5.11) 🡪 Y(1.11) | 14.950 |
|  |  | A(5.11) 🡪 Z(2.11) | 09.042 |
|  |  | A(5.11) 🡪 Z(3.11) | 14.337 |
|  |  | A(5.11) 🡪 H(4.11) | 16.725 |
|  |  |  |  |
| 10 | Y(1.11) Z(2.11) Z(3.11) H(4.11) A(5.11)  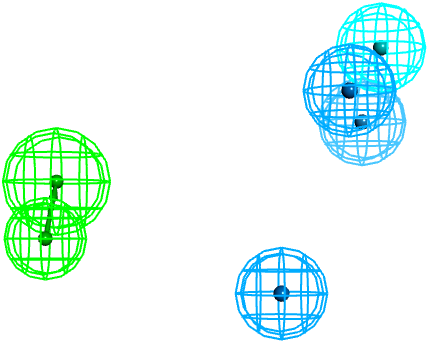 | Y(1.11) 🡪 Z(2.11) | 03.359 |
|  |  | Y(1.11) 🡪 Z(3.11) | 07.904 |
|  |  | Y(1.11) 🡪 H(4.11) | 03.054 |
|  |  | H(4.11) 🡪 Z(2.11) | 02.964 |
|  |  | H(4.11) 🡪 Z(3.11) | 10.211 |
|  |  | Z(2.11) 🡪 Z(3.11) | 07.889 |
|  |  | A(5.11) 🡪 Y(1.11) | 13.891 |
|  |  | A(5.11) 🡪 Z(2.11) | 14.448 |
|  |  | A(5.11) 🡪 Z(3.11) | 11.221 |
|  |  | A(5.11) 🡪 H(4.11) | 15.772 |

**Supplementary table 2:** The list of the test-set validation parameters for the allosteric site binded inhibitors.

| Total Actives: **62** | | | | | | | |
| --- | --- | --- | --- | --- | --- | --- | --- |
| Total Inactives: **221** | | | | | | | |
| **Model** | **True Positives** | **True Negatives** | **False Positives** | **False Negatives** | **Sensitivity** | **Specificity** | **AUC-ROC** |
| **M1** | 15 | 219 | 2 | 47 | 0.24 | 0.99 | 0.62 |
| **M2** | 20 | 219 | 2 | 42 | 0.32 | 0.99 | 0.66 |
| **M3** | 19 | 219 | 2 | 43 | 0.31 | 0.99 | 0.65 |
| **M4** | 07 | 221 | 0 | 55 | 0.11 | 1.00 | 0.56 |
| **M5** | 12 | 221 | 0 | 50 | 0.19 | 1.00 | 0.60 |
| **M6** | 15 | 221 | 0 | 47 | 0.24 | 1.00 | 0.62 |
| **M7** | 14 | 220 | 1 | 48 | 0.23 | 0.99 | 0.61 |
| **M8** | 15 | 221 | 0 | 47 | 0.24 | 1.00 | 0.62 |
| **M9** | 06 | 221 | 0 | 56 | 0.10 | 1.00 | 0.55 |
| **M10** | 05 | 221 | 0 | 57 | 0.08 | 1.00 | 0.54 |
| Total Actives: **62** | | | | | | | |
| Total Inactives: **3895** | | | | | | | |
| **Model** | **True Positives** | **True Negatives** | **False Positives** | **False Negatives** | **Sensitivity** | **Specificity** | **AUC-ROC** |
| **M1** | 15 | 3624 | 271 | 47 | 0.24 | 0.93 | 0.58 |
| **M2** | 20 | 3645 | 250 | 42 | 0.32 | 0.94 | 0.62 |
| **M3** | 19 | 3618 | 277 | 43 | 0.31 | 0.93 | 0.61 |
| **M4** | 7 | 3852 | 43 | 55 | 0.11 | 0.99 | 0.55 |
| **M5** | 12 | 3766 | 129 | 50 | 0.19 | 0.97 | 0.58 |
| **M6** | 15 | 3746 | 149 | 47 | 0.24 | 0.96 | 0.60 |
| **M7** | 14 | 3756 | 139 | 48 | 0.23 | 0.96 | 0.59 |
| **M8** | 15 | 3745 | 150 | 47 | 0.24 | 0.96 | 0.60 |
| **M9** | 6 | 3762 | 133 | 56 | 0.10 | 0.97 | 0.53 |
| **M10** | 5 | 3754 | 141 | 57 | 0.08 | 0.96 | 0.52 |


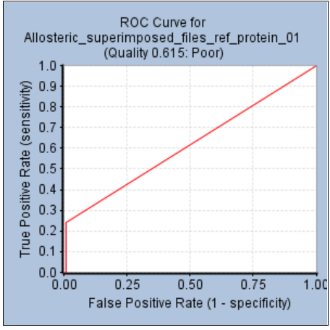

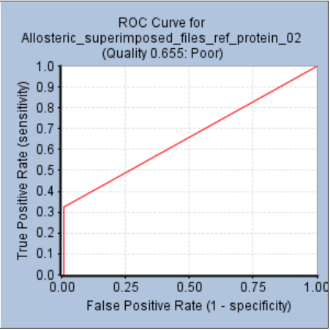

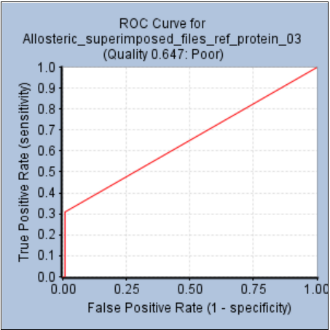

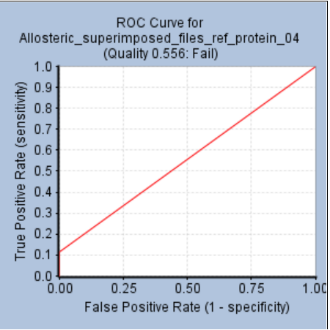

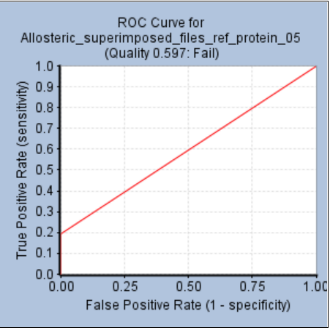

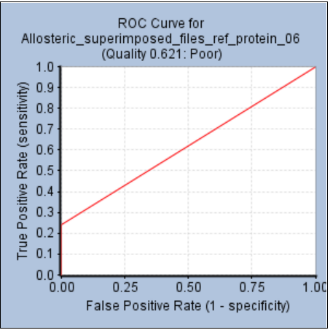

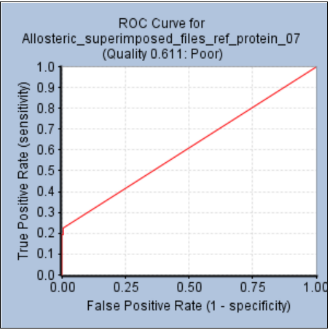

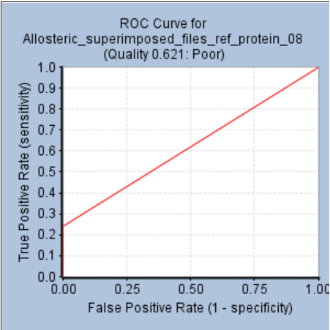

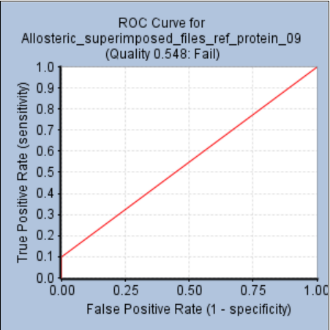

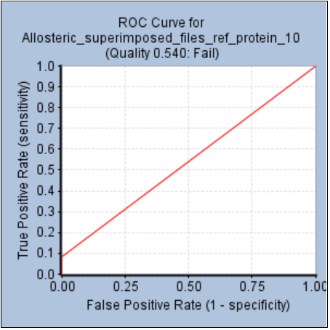


**Supplementary Figure 1:** Pictorial representation of ROC-AUC plots obtained from ten pharmacophores generated from allosteric-bonded inhibitors of MTHFD2 protein.

**Supplementary table 3:** List of EF and GH scoring parameters for the validation of the pharmacophore models.

| Total molecules in database (D): **796** | | | | | | | | |
| --- | --- | --- | --- | --- | --- | --- | --- | --- |
| Total number of experimental actives in database (A): **15** | | | | | | | | |
| **Model** | **Total Hits**  **(Ht)** | **Active Hits**  **(Ha)** | **% Yield of actives [(Ha/Ht)×100]** | **% Ratio of actives [(Ha/A)×100]** | **Enrichment factor [(Ha×D)/(Ht×A)]** | **False negatives [A-Ha]** | **False positives [Ht-Ha]** | **Goodness of hit score** |
| **M1** | 19 | 02 | 010.53 | 13.33 | 05.59 | 13.00 | 17.00 | 0.091 |
| **M2** | 16 | 02 | 012.50 | 13.33 | 06.63 | 13.00 | 14.00 | 0.109 |
| **M3** | 20 | 02 | 010.00 | 13.33 | 05.31 | 13.00 | 18.00 | 0.085 |
| **M4** | 01 | 01 | 100.00 | 06.67 | 53.07 | 14.00 | 00.00 | 0.767 |
| **M5** | 03 | 02 | 066.67 | 13.33 | 35.38 | 13.00 | 01.00 | 0.532 |
| **M6** | 04 | 02 | 050.00 | 13.33 | 26.53 | 13.00 | 02.00 | 0.406 |
| **M7** | 04 | 02 | 050.00 | 13.33 | 26.53 | 13.00 | 02.00 | 0.406 |
| **M8** | 05 | 02 | 040.00 | 13.33 | 21.23 | 13.00 | 03.00 | 0.329 |
| **M9** | 02 | 00 | 000.00 | 00.00 | 00.00 | 15.00 | 02.00 | -0.003 |
| **M10** | 02 | 00 | 000.00 | 00.00 | 00.00 | 15.00 | 02.00 | -0.003 |

**Supplementary table 4:** List of the number of molecules screened from COCONUT, Drug-like Diverse, Specs, and UNPD database from the respective generated pharmacophores.

| **Model Number** | **Features** | **Number of molecules screened** |
| --- | --- | --- |
| 01 | YZZAA | **195**  [COCO = 18, DDD = 64, SPECS = 35, UNPD = 78] |
| 02 | RZZAA | **108**  [COCO = 13, DDD = 40, SPECS = 18, UNPD = 37] |
| 03 | RZZAA | **206**  [COCO = 19, DDD = 64, SPECS = 25, UNPD = 90] |
| 04 | YZZHD | **032**  [COCO = 08, DDD = 03, SPECS = 10, UNPD = 11] |
| 05 | RZZHD | **073**  [COCO = 09, DDD = 07, SPECS = 08, UNPD = 49] |
| 06 | RZZHD | **092**  [COCO = 10, DDD = 18, SPECS = 11, UNPD = 53] |
| 07 | ZZHHA | **252**  [COCO = 132, DDD = 07, SPECS = 60, UNPD = 53] |
| 08 | YZZHA | **095**  [COCO = 16, DDD = 22, SPECS = 26, UNPD = 31] |
| 09 | YZZHA | **094**  [COCO = 15, DDD = 23, SPECS = 25, UNPD = 31] |
| 10 | YZZHA | **074**  [COCO = 17, DDD = 17, SPECS = 20, UNPD = 20] |
| COCO: COCONUT Database, DDD: Drug-like Diverse Database, UNPD: Universal Natural Product Database | | |


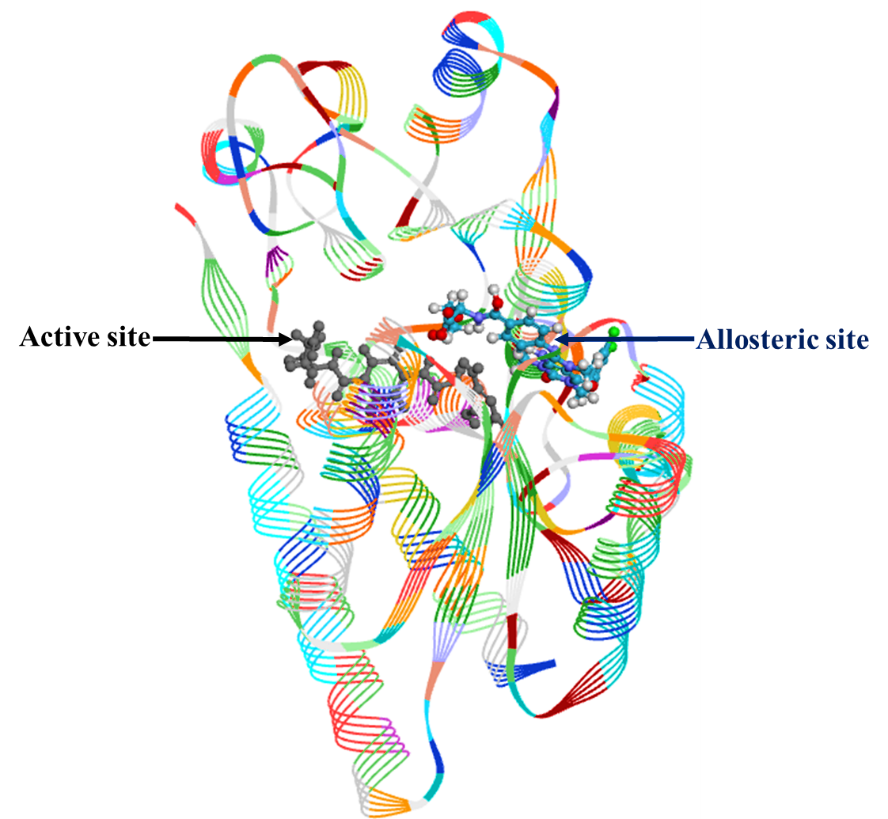


**Supplementary Figure 2:** Pictorial representation of the MTHFD2 protein of PDB ID 7EHM co-crystallised with two inhibitors, *i.e.*, J49 and J4C. Inhibitor J49 and J4C is co-crystallised in the active and allosteric binding site of the protein respectively.

**
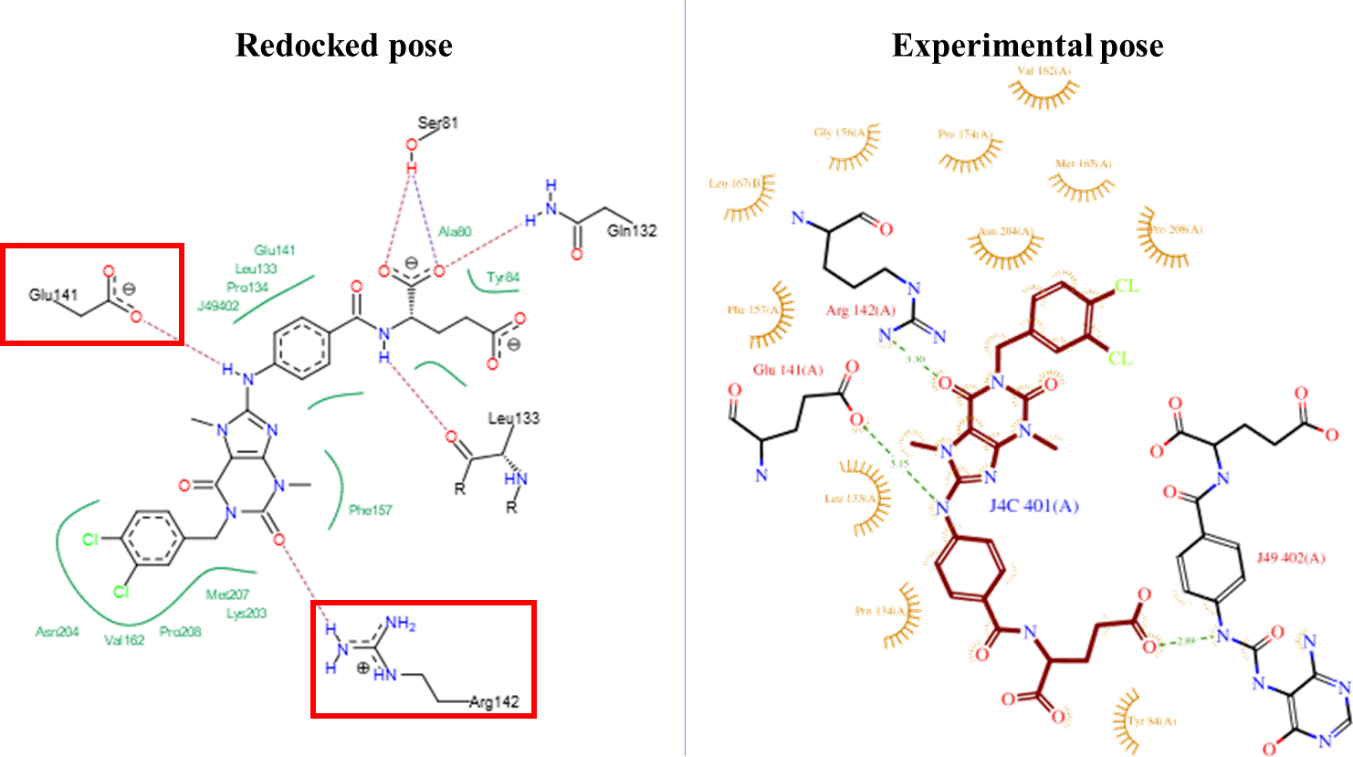
**

**Supplementary Figure 3:** 2D interaction plot of the redocked and experimentally co-crystallised pose of J4C of PDB ID 7EHM. Red box on left side indicates the interaction with the crucial amino acids, as reported in the experimental pose.

**Supplementary table 5:** List of the docking score along with the HYDE assessment parameters (free energy and ligand efficiency) of the docked natural product candidates.

| **Pose name** | **Docking score** | **∆G**  **(KJ/mol)** | **LE (mM)** | **Pose name** | **Docking score** | **∆G**  **(KJ/mol)** | **LE (mM)** |
| --- | --- | --- | --- | --- | --- | --- | --- |
| dinitroanilino derivative | -35.19 | -13 | 0.08 | ENA363437 | -19.53 | -22 | 0.19 |
| Aminodeacetoxy derivative | -33.71 | 04 | 00 | ENA735644 | -19.52 | -09 | 0.07 |
| CDI903213 | -32.08 | -17 | 0.14 | UNPD162518 | -19.41 | -16 | 0.14 |
| UNPD7949 | -31.04 | -14 | 0.12 | ASI1136988 | -19.38 | -01 | 0.01 |
| CNP0000225 | -30.97 | -10 | 0.06 | Hespiridin | -19.34 | -09 | 0.05 |
| UKR603097 | -30.79 | 10 | 00 | UNPD198940 | -19.27 | -24 | 00 |
| CNP0000022 | -30.73 | -29 | 0.17 | CDI385803 | -19.24 | -16 | 0.17 |
| CDI727753 | -30.59 | -18 | 0.13 (µM) | IBS413356 | -19.23 | -04 | 0.04 |
| ENA1231127 | -28.77 | -30 | 0.23 | UNPD210711 | -19.18 | -04 | 0.04 |
| CNP0000223 | -28.72 | -23 | 0.10 | UNPD165992 | -19.17 | -04 | 0.04 |
| UNPD214408 | -28.70 | -14 | 0.13 | * UKR1227978 | -19.17 | -29 | 0.22 (µM) |
| UNPD216129 | -28.64 | -13 | 0.11 | CBG587174 | -19.06 | -03 | 0.02 |
| CDI819489 | -28.54 | -08 | 0.06 | * UNPD28642 | -18.99 | -27 | 0.22 (µM) |
| UKR53126 | -28.46 | 06 | 00 | * UNPD212860 | -18.88 | -24 | 0.21 (µM) |

**Supplementary table 5: Continued……**

| **Pose name** | **Docking score** | **∆G**  **(KJ/mol)** | **LE (mM)** | **Pose name** | **Docking score** | **∆G**  **(KJ/mol)** | **LE (mM)** |
| --- | --- | --- | --- | --- | --- | --- | --- |
| ENA1283391 | -28.41 | -03 | 0.02 | 3-[(1,3-dioxo-1,3-dihydro-2H-isoindol) derivative | -18.84 | -04 | 0.02 |
| CNP0000222 | -28.40 | -24 | 0.12 | * UKR1475977 | -18.84 | -22 | 0.21 (µM) |
| CBG91227 | -28.23 | -07 | 0.05 | * UNPD114808 | -18.82 | -17 | 0.21 |
| UNPD112926 | -27.99 | -30 | 0.28  (µM) | Cnidimoside | -18.67 | -18 | 0.14 |
| CDI909983 | -27.58 | -03 | 0.02 | UNPD150296 | -18.66 | -15 | 0.14 |
| UNPD177339 | -27.45 | -15 | 0.13 | VIT924036 | -18.66 | -20 | 0.16 |
| ASI306827 | -27.44 | -22 | 0.19 | * UNPD224158 | -18.59 | -22 | 0.21 |
| ethyl 3-(3,4- dimethoxyphenyl) derivative | -27.38 | -10 | 0.05 | UNPD113239 | -18.58 | -03 | 0.03 |
| *CBG223815 | -27.34 | -39 | 0.25  (nM) | UNPD217979 | -18.57 | -12 | 0.10 |
| VIT415811 | -27.28 | -21 | 0.17 | UNPD221688 | -18.47 | -09 | 0.08 |
| Lantibeside | -27.26 | -34 | 0.17  (µM) | UNPD143116 | -18.45 | -09 | 0.08 |
| UNPD146880 | -26.92 | -05 | 0.05 | UNPD222437 | -18.42 | -07 | 0.06 |
| UKR1329295 | -26.92 | -10 | 0.08 | UNPD46657 | -18.39 | 04 | 00 |
| UKR574365 | -26.77 | -03 | 0.02 | UNPD202888 | -18.38 | -20 | 0.17 |
| ANC5908 | -26.70 | -31 | 0.19  (µM) | CNP0000028 | -18.35 | -17 | 0.10 |
| CDI621734 | -26.64 | -22 | 0.15  (µM) | * UNPD151821 | -18.33 | -31 | 0.28 (µM) |
| CNP0000224 | -26.26 | 18 | 00 | * CNP0000287 | -18.30 | -27 | 0.2 (µM) |
| N-carbamoyl-Doxorubicin | -26.24 | 07 | 00 | * UNPD230017 | -18.26 | -24 | 0.21 (µM) |
| CDI807283 | -26.05 | -18 | 0.12 | CD1703283 | -18.26 | -06 | 0.05 |
| UNPD224830 | -26.01 | 17 | 00 | UKR347099 | -18.26 | -08 | 0.07 |
| UNPD204453 | -25.89 | -01 | 0.01 | UKR881834 | -18.25 | -19 | 0.15 |
| CNP0000004 | -25.63 | 17 | 00 | UNPD110129 | -18.13 | -14 | 0.12 |
| UKR35326 | -25.59 | 03 | 00 | * UNPD7184 | -17.98 | -36 | 0.28 (nM) |
| CDI685470 | -25.35 | -08 | 0.05 | UNPD155834 | -17.96 | -04 | 0.03 |
| * UNPD222976 | -25.33 | -23 | 0.2  (µM) | * ENA259832 | -17.93 | -23 | 0.24 |
| PEK6887 | -25.29 | -16 | 0.12 | UNPD194478 | -17.88 | -19 | 0.16 |
| UKR266793 | -25.14 | -14 | 0.11 | Methyl 9-(5,,7-dimethoxy derivative | -17.80 | -19 | 0.13 |
| CDI865703 | -25.06 | -20 | 0.14 | * UNPD50612 | -17.75 | -25 | 0.20 |
| UKR373341 | -24.92 | -05 | 0.09 | CNP0000289 | -17.73 | -16 | 0.12 |
| CDI883466 | -24.74 | 00 | 00 | UNPD104415 | -17.69 | -11 | 0.09 |

**Supplementary table 5: Continued……**

| **Pose name** | **Docking score** | **∆G**  **(KJ/mol)** | **LE (mM)** | **Pose name** | **Docking score** | **∆G**  **(KJ/mol)** | **LE (mM)** |
| --- | --- | --- | --- | --- | --- | --- | --- |
| CDI484583 | -24.64 | -25 | 0.17  (µM) | UNPD74238 | -17.67 | -33 | 0.27 (µM) |
| Tecomelloside | -24.53 | -12 | 0.08 | PHA106751 | -17.68 | 07 | 00  (µM) |
| SPE115069 | -24.52 | -21 | 0.19  (µM) | UNPD40361 | -17.52 | -04 | 00 (µM) |
| IBS405336 | -24.46 | -22 | 0.15 | UNPD101160 | -17.51 | -31 | 0.20 (µM) |
| ENA152165 | -24.44 | -22 | 0.17 | UKR210657 | -17.51 | -21 | 0.17 (µM) |
| UKR158413 | -24.20 | 08 | 00 | UNPD224306 | -17.50 | -01 | 0.07 (µM) |
| UKR912707 | -24.16 | 09 | 00 | UNPD144077 | -17.50 | -13 | 0.09 (µM) |
| * UNPD163581 | -24.08 | -22 | 0.21 | UNPD152795 | -17.47 | -02 | 0.02 (µM) |
| UNPD136713 | -24.05 | -23 | 0.18 | UNPD222354 | -17.43 | -13 | 0.09 (µM) |
| * CAP05036418 | -24.04 | -28 | 0.27  (µM) | CNP0000017 | -17.40 | -24 | 0.13 (µM) |
| CNP0000018 | -23.93 | -15 | 0.11 | UNPD175981 | -17.40 | -11 | 0.10 |
| UNPD198245 | -23.88 | 06 | 00 | UNPD167061 | -17.36 | -21 | 0.19 |
| * ENA41862 | -23.85 | -30 | 0.24  (µM) | CBG98066 | -17.32 | -10 | 0.08 |
| ENA153647 | -23.85 | -06 | 0.04 | ENA305490 | -17.29 | -01 | 0.01 |
| CBG57602 | -23.79 | -07 | 0.06 | UNPD201442 | -17.29 | -01 | 0.01 |
| UKR770996 | -23.73 | -25 | 0.18 | UNPD152731 | -17.28 | -14 | 0.11 |
| CBG514261 | -23.62 | -08 | 0.07 | UNPD164095 | -17.24 | 05 | 00 |
| UNPD158106 | -23.61 | -18 | 0.17 | UNPD132258 | -17.19 | -17 | 0.14 |
| UNPD230088 | -23.56 | -13 | 0.13 | UNPD205233 | -17.13 | -15 | 0.10 |
| * ASI311667 | -23.48 | -33 | 0.27  (µM) | UNPD203167 | -17.10 | -01 | 0.01 |
| * UNPD212047 | -23.46 | -28 | 0.26  (µM) | Gambogic acid | -17.09 | -20 | 0.10 |
| * UNPD115741 | -23.45 | -26 | 0.22  (µM) | UNPD187356 | -17.09 | -17 | 0.15 |
| * PHA170542 | -23.41 | -24 | 0.23  (µM) | UNPD49446 | -17.07 | -22 | 0.18 |
| IBS432164 | -23.39 | -15 | 0.12 | UNPD23185 | -17.04 | -21 | 0.18 |
| CBG157961 | -23.33 | -17 | 0.15 | CNP0000003 | -16.98 | -20 | 0.11 |
| CAP00252078 | -23.32 | 02 | 00 | *UNPD229253 | -16.85 | -20 | 0.20 (µM) |
| CNP0000219 | -23.30 | -12 | 0.07 | UNPD112504 | -16.84 | -19 | 0.18 |
| ENA1099689 | -23.07 | -21 | 0.18 | UNPD203166 | -16.78 | -05 | 0.04 |
| * ENA381373 | -23.02 | -32 | 0.27  (µM) | UKR961802 | -16.75 | -36 | 0.29 |
| * CDI352052 | -23.01 | -26 | 0.26  (µM) | *UNPD203182 | -16.72 | -38 | 0.34 |
| * CBG512889 | -23.01 | -26 | 0.21  (µM) | UNPD208415 | -16.65 | -03 | 0.03 |
| CDI5733 | -22.96 | 26 | 00 | Evoxine acetate | -16.62 | -06 | 0.05 |
| Embelin derivatives | -22.91 | 08 | 00 | Haplopine derivative | -16.52 | -13 | 0.09 |
| * UNPD69504 | -22.90 | -38 | 0.33  (nM) | UNPD196095 | -16.36 | -01 | 0.01 |

**Supplementary table 5: Continued……**

| **Pose name** | **Docking score** | **∆G**  **(KJ/mol)** | **LE (mM)** | **Pose name** | **Docking score** | **∆G**  **(KJ/mol)** | **LE (mM)** |
| --- | --- | --- | --- | --- | --- | --- | --- |
| SPE81873 | -22.89 | -21 | 0.17 | UNPD176267 | -16.35 | -13 | 0.11 |
| CDI94805 | -22.88 | 01 | 00 | CNP0000281 | -16.15 | -54 | 0.28 |
| ENA1095030 | -22.81 | 11 | 00 | UNPD203869 | -16.08 | -17 | 0.12 |
| CD1590679 | -22.69 | -11 | 0.08  (nM) | UNPD117452 | -16.05 | -10 | 0.07 |
| LIF212943 | -22.67 | -21 | 0.15 | UNPD142475 | -16.04 | -12 | 0.10 |
| UNPD154407 | -22.57 | -20 | 0.20 | UNPD153894 | -16.02 | -37 | 0.32 |
| ASI194107 | -22.52 | -11 | 0.09 | UNPD193410 | -16.01 | -08 | 0.07 |
| * UNPD91301 | -22.47 | -20 | 0.20  (µM) | UNPD111756 | -15.95 | -13 | 0.11 |
| UNPD176783 | -22.46 | -06 | 0.05 | CNP0000295 | -15.91 | -27 | 0.23 |
| UNPD200548 | -22.44 | -21 | 0.15 | UNPD172481 | -15.88 | -16 | 0.13 |
| UNPD122095 | -22.42 | -17 | 0.14 | UNPD228656 | -15.87 | -18 | 0.14 |
| UNPD199045 | -22.41 | 06 | 00 | UNPD163875 | -15.74 | -01 | 0.01 |
| CBG419470 | -22.41 | -18 | 0.15 | UKR881884 | -15.72 | -08 | 0.06 |
| UKR211504 | -22.24 | -07 | 0.06 | UNPD133305 | -15.69 | -13 | 0.10 |
| UNPD108730 | -22.23 | -04 | 0.03 | UNPD80004 | -15.65 | -11 | 0.09 |
| UNPD43402 | -22.21 | -04 | 0.04 | UNPD218004 | -15.63 | -24 | 0.18 |
| * UNPD197265 | -22.15 | -22 | 0.21  (µM) | ASI189605 | -15.59 | -12 | 0.10 |
| * UNPD53737 | -22.11 | -38 | 0.32  (nM) | UNPD3074 | -15.41 | -29 | 0.22 |
| * UNPD224655 | -22.10 | -28 | 0.26  (µM) | Thalicarpine | -15.26 | -25 | 0.12 |
| * UNPD167177 | -22.09 | -28 | 0.26  (µM) | UNPD202303 | -15.25 | -28 | 0.28 |
| UNPD92924 | -22.09 | -10 | 0.09 | CBG201279 | -15.19 | -10 | 0.08 |
| CDI171686 | -22.08 | -17 | 0.14 | UNPD105960 | -14.98 | -19 | 0.16 |
| ENA728442 | -22.07 | -07 | 0.05 | UNPD141654 | -14.91 | -14 | 0.12 |
| Silybin derivative | -22.06 | -03 | 0.02 | UNPD87153 | -14.82 | -32 | 0.25 |
| UNPD164701 | -22.02 | -17 | 0.15 | UNPD7609 | -14.79 | -32 | 0.26 |
| UNPD179445 | -22.01 | -23 | 0.19 | UNPD10142 | -14.72 | -14 | 0.13 |
| CDI706546 | -21.99 | -15 | 0.10 | CNP0000247 | -14.68 | -19 | 0.09 |
| * UNPD217941 | -21.94 | -38 | 0.31  (nM) | CNP0000188 | -14.56 | -14 | 0.13 |
| CDI904370 | -21.86 | 06 | 00 | CNP0000248 | -14.55 | -14 | 0.07 |
| CNP0000288 | -21.85 | -23 | 0.19 | UNPD205341 | -14.50 | -14 | 0.11 |
| * CBG214922 | -21.79 | -26 | 0.2  (µM) | UNPD121244 | -14.49 | 01 | 00 |
| UNPD216056 | -21.77 | -14 | 0.13 | UNPD48326 | -14.38 | -05 | 0.04 |
| Amorphin | -21.76 | -02 | 0.01 | UNPD5 | -14.36 | -18 | 0.14 |
| UNPD18679 | -21.72 | -13 | 0.12 | UNPD211242 | -14.32 | -37 | 0.30 |
| ENA241749 | -21.68 | -16 | 0.13 | CNP0000283 | -14.03 | -15 | 0.14 |
| UKR1333851 | -21.63 | -12 | 0.10 | CNP0000265 | -13.97 | -10 | 0.05 |
| ENA168582 | -21.61 | 01 | 00 | UNPD30748 | -13.89 | -26 | 0.23 (µM) |
| CDI710842 | -21.61 | -16 | 0.11 | UNPD15083 | -13.88 | -21 | 0.14 (µM) |
| CDI447084 | -21.57 | 04 | 00 | UNPD202300 | -13.78 | -14 | 0.14 |

**Supplementary table 5: Continued……**

| **Pose name** | **Docking score** | **∆G**  **(KJ/mol)** | **LE (mM)** | **Pose name** | **Docking score** | **∆G**  **(KJ/mol)** | **LE (mM)** |
| --- | --- | --- | --- | --- | --- | --- | --- |
| CNP0000220 | -21.51 | -16 | 0.09 | UNPD99607 | -13.59 | -40 | 0.35 (nM) |
| * UNPD204731 | -21.39 | -28 | 0.27  (µM) | UNPD195858 | -13.58 | -29 | 0.21 (µM) |
| UNPD208379 | -21.32 | -02 | 0.01 | Colchamine derivative | -13.46 | -02 | 0.01 |
| ENA265368 | -21.31 | -03 | 0.02 | UNPD59491 | -13.09 | -11 | 0.08 |
| CAP00244134 | -21.24 | -14 | 0.14 | UNPD159440 | -12.96 | -29 | 0.27 |
| ENA1018719 | -21.14 | -10 | 0.08 | CNP0000184 | -12.74 | -19 | 0.17 |
| * UNPD155889 | -21.13 | -25 | 0.23  (µM) | UNPD146017 | -12.69 | 17 | 00 |
| TIM84173 | -21.11 | -13 | 0.11 | UNPD144149 | -12.35 | 09 | 00 |
| * UNPD230014 | -21.09 | -39 | 0.35  (nM) | Dehydrothalicarpin | -11.69 | -18 | 0.09 |
| CNP0000221 | -21.08 | -16 | 0.10 | UNPD135113 | -11.64 | -18 | 0.14 |
| CBG24095 | -21.08 | -17 | 0.11 | UNPD33316 | -11.62 | -20 | 0.15 |
| Icariin | -20.95 | -20 | 0.10 | UNPD2489 | -11.58 | -12 | 0.08 |
| UNPD222925 | -20.90 | -19 | 0.18 | UNPD147346 | -11.44 | -07 | 0.05 |
| UNPD142635 | -20.84 | -20 | 0.16 | UNPD212526 | -10.94 | -38 | 0.26 (nM) |
| UKR1037467 | -20.83 | -21 | 0.19 | CNP0000277 | -10.81 | -12 | 0.09 |
| * UNPD26285 | -20.79 | -31 | 0.30  (µM) | UNPD220697 | -10.68 | -26 | 0.18 |
| ENA1126321 | -20.78 | -05 | 0.04 | Caephaline derivative | -10.68 | -15 | 0.09 |
| * UNPD221725 | -20.78 | -32 | 0.27  (µM) | UNPD202301 | -10.66 | -17 | 0.17 |
| Neferine | -20.77 | -23 | 0.12  (µM) | UNPD53792 | -10.38 | -02 | 0.01 |
| * UNPD224603 | -20.71 | -23 | 0.21  (µM) | CNP0000043 | -10.30 | -38 | 0.25 (nM) |
| UNPD206221 | -20.70 | -19 | 0.19  (µM) | UNPD96304 | -9.94 | -34 | 0.24 (µM) |
| UNPD4105 | -20.69 | -20 | 0.18  (µM) | CNP0000276 | -9.79 | -02 | 0.01 |
| UNPD213851 | -20.68 | -14 | 0.13 | UNPD53078 | -9.76 | -08 | 0.05 |
| UNPD129173 | -20.67 | -14 | 0.13 | 2,5-Dihydroxy derivative | -9.29 | -32 | 0.31 (µM) |
| UNPD194802 | -20.67 | -09 | 0.08 | Caephaline derivative | -9.08 | -04 | 0.02 |
| UKR291623 | -20.61 | -22 | 0.19 | UNPD177137 | -8.36 | -36 | 0.24 (µM) |
| UNPD133082 | -20.54 | 01 | 00 | UNPD24554 | -7.21 | -16 | 0.11 |
| UNPD193879 | -20.52 | -18 | 0.16 | UNPD38034 | -7.15 | -27 | 0.19 |
| * Tirunesiin | -20.44 | -32 | 0.24 | Dihydromaesanin | -6.78 | -18 | 0.16 |
| UKR1345557 | -20.43 | -18 | 0.15 | UNPD123873 | -6.5126 | -9 | 0.06 |
| UNPD16273 | -20.42 | -08 | 0.07 | UNPD51103 | -6.29 | -09 | 0.06 |
| UNPD113476 | -20.41 | -15 | 0.12 | Tyromycin | -6.12 | -09 | 0.07 |
| CD1453733 | -20.29 | -09 | 0.07 | Maesaquinone | -5.99 | -20 | 0.16 |
| UNPD203806 | -20.23 | -15 | 0.12 | CNP0000292 | -5.69 | -19 | 0.11 |
| * ENA245097 | -20.22 | -30 | 0.27 | 3,20,29-Lupanetriol | -4.10 | -09 | 0.05 |
| UNPD116702 | -20.13 | -08 | 0.07 | Maesaquinone Dimethyl ether | -3.62 | -33 | 0.25 |

**Supplementary table 5: Continued……**

| **Pose name** | **Docking score** | **∆G**  **(KJ/mol)** | **LE (mM)** | **Pose name** | **Docking score** | **∆G**  **(KJ/mol)** | **LE (mM)** |
| --- | --- | --- | --- | --- | --- | --- | --- |
| Picroside II | -20.13 | -10 | 0.07 | Thalimelatine | -3.37 | -19 | 0.09 |
| UNPD8406 | -20.10 | -12 | 0.09 | Dihydromaesaquinon | -3.26 | -43 | 0.34 (nM) |
| UNPD25164 | -20.07 | -24 | 0.18 | CNP0000305 | -2.89 | -11 | 0.11 |
| CDI625549 | -20.07 | -15 | 0.11 | Oleanoic acid derivative | -2.88 | -11 | 00 |
| UNPD12024 | -19.96 | -04 | 0.03 | UNPD10517 | -2.75 | -14 | 0.10 |
| UNPD129267 | -19.95 | -21 | 0.17 | Maesaquinone diacetate | -2.22 | -45 | 0.30 (nM) |
| Buddleoflavonoloside | -19.92 | -06 | 0.03 | Crinosterol derivative | -1.31 | -28 | 0.20 (µM) |
| UNPD228600 | -19.91 | -19 | 0.17 | UNPD12649 | -1.29 | -18 | 0.13 |
| * OTA438 | -19.89 | -52 | 0.20 | UNPD212845 | -1.23 | -34 | 0.24 (µM) |
| * UKR1361991 | -19.86 | -26 | 0.21 | Beesioside I, derivative | 0.52 | 03 | 00 |
| UNPD12178 | -19.84 | -10 | 0.09 | Uvaol diacetate | 0.96 | -04 | 0.02 |
| UNPD21948 | -19.81 | -10 | 0.08 | UNPD227913 | 2.25 | -16 | 0.11 |
| UNPD47989 | -19.74 | -05 | 0.04 | Deucosterol | 2.53 | -12 | 0.07 |
| ENA954498 | -19.70 | -05 | 0.04 | Astramembrannin | 3.68 | -09 | 0.05 |
| UNPD20645 | -19.67 | -03 | 0.02 | UNPD116717 | 5.50 | -20 | 0.14 |
| * UNPD103789 | -19.66 | -22 | 0.21 | Naringenin Derivative | 11.12 | 14 | 00 |
| UNPD183879 | -19.55 | -16 | 0.15 |  |  |  |  |

**Supplementary table 6:** List of the molecules filtered via Lipinski rule of five (RO5), Veber rule and ADMET parameters.

| **S. No.** | **Molecule^*^** | **ADMET Solubility Level^**^** | **ADMET BBB Level^$^** | **ADMET PSA 2D^Δ^** | **ADMET Alog*P*98** | **Interacting amino acids** |
| --- | --- | --- | --- | --- | --- | --- |
| 01 | UNPD112926 | 3 | 3 | 089.236 | 2.188 | **Ser81, Gln132, Leu133, Asn204, Val274,** Ala80, Tyr84, Thr176, Val205, Ile276, J49402 |
| 02 | CBG223815 | 3 | 3 | 079.820 | 2.408 | Leu133, Phe157, Thr176, Asn204, Val205, Val274, Ile276, J49402 |
| 03 | UNPD222976 | 3 | 3 | 103.022 | 0.948 | **Asn204, Ile276,** Phe157, Asn204, Val205, Ile276, J49402 |
| 04 | UNPD163581 | 3 | 3 | 089.236 | 2.188 | **Asn204, Ile276,** Phe157, Thr176, Asn204, Val205, Val274, Ile276, J49402 |
| 05 | ASI311667^#^ | 3 | 2 | 029.994 | 1.344 | **Glu141, Asn204, Leu133**, Tyr84, Leu133, Pro134, Glu141, Phe157, Thr176, Asn204, Val205, Ile276, J49402 |
| 06 | UNPD212047 | 3 | 3 | 089.236 | 2.188 | **Asn204, Ile276,** Phe157, Thr176, Val205, Val274, Ile276, J49402 |
| 07 | UNPD69504 | 3 | 3 | 081.648 | 2.769 | **Asn204,** Phe157, Asn204, Val205, Leu133, Val162, J49402 |
| 08 | UNPD224655^#^ | 3 | 3 | 085.722 | 2.444 | **Glu141, Asn204, Ile276,** Phe157, Leu133, Val205, Ile276, J49402 |
| 09 | UNPD167177 | 3 | 3 | 085.722 | 2.444 | **Glu141, Asn204, Ile276,** Phe157, Leu133, Val205, Ile276, J49402 |
| 10 | UNPD155889 | 3 | 3 | 089.236 | 2.855 | **Ser81, Gln132, Leu133, Ile276,** Pro134, Tyr84, Ile276, Ala80, Leu133, J49402 |
| 11 | UNPD230014^#^ | 3 | 3 | 106.537 | 2.220 | **Glu141, Asn204, Ile276,** Asn204, Thr176, Val205, Val274, Phe157, Leu133, J49402 |
| 12 | UNPD221725 | 3 | 3 | 091.137 | 2.823 | Leu133, Arg142, Cys145, Phe157, Thr176, Asn204, Val205, J49402 |
| 13 | UNPD224603^#^ | 3 | 3 | 089.236 | 2.188 | **Glu141, Asn204, Val274, Ile276,** Leu133, Phe157, Asn204, Val205, Ile276, J49402 |
| 14 | UNPD151821^#^ | 3 | 3 | 103.321 | 2.362 | **Gln132, Leu133, Glu141, Asn204,** Ala80, Tyr84, Leu133, Asn204, Val205, Ile276, J49402 |
| 15 | UNPD230017 | 3 | 3 | 098.166 | 2.257 | **Thr176, Asn204, Val274,** Phe157, Pro174, Thr176, Pro177, Val205, Pro208, Thr316, J49402 |
| 16 | UNPD74238 | 3 | 3 | 081.648 | 2.803 | **Asn204,** Leu133, Phe157, Val162, Asn204, Val205, J49402 |
| 17 | UNPD101160^#^ | 3 | 3 | 098.166 | 3.211 | **Glu141, Arg142, Asn204,** Tyr84, Leu133, Glu141, Arg142, Phe157, Thr176, Asn204, Val205, Ile276, J49402 |
| 18 | UNPD203074 | 3 | 3 | 097.607 | 2.849 | **Thr176,** Phe157, Val162, Pro174, Pro177, Thr176, Pro177, Asn204, Val205, Pro208, J49402 |
| 19 | UNPD202303^#^ | 3 | 3 | 081.648 | 2.832 | **Glu141, Arg142, Asn204,** Leu133, Glu141, Phe157, Asn204 |
| 20 | UNPD159440 | 3 | 3 | 085.722 | 2.956 | **Asn204, Ile276,** Leu133, Phe157, Thr176, Asn204, Val205, Ile276, J49402 |
| ^*^All 20 molecules: follow Lipinski RO5 and Veber rule, CYP2D6 prediction (FALSE), Hepatotoxicity prediction (FALSE), PPB prediction (True) and Adsorption level (0).  ^**^ADMET solubility level: 0- extremely low, 1- very low but possible, 2- low, 3- good, 4- optimal and 5- too soluble, 6- warning, molecule with one or more unknown AlogP98 types.  ^$^ADMET BBB level: 0- very high penetrant, 2- medium penetrant, 3- low penetrant and 4-undefined.  ^Δ^ADMET PSA: <140  ADMET adsorption level: 0- good absorption, 1- moderate absorption, 2- poor absorption and 3- very poor, Absorption, ADMET CYP2D6, ADMET Hepatotoxic, and ADMET PPB: from Bayesian statistics.  ^#^Selected for conducting simulation studies | | | | | | |

**Supplementary table 7:** List of the docking score, HYDE assessed-binding affinity (**∆G**), and ligand efficiency (**∆E**) of the top six shortlisted natural product molecules against MTHFD2 allosteric site.

| **Molecule** | **IUPAC name** | **Docking Score**  **(kcal/mol)** | **HYDE assessment** | |
| --- | --- | --- | --- | --- |
|  |  |  | **∆G (kcal/mol)** | **∆E (µM)** |
| ASI311667 | N-(2-((2,4-dichlorobenzyl)oxy)-3-methoxybenzyl)-3-morpholinopropan-1-amine | -23.48 | -33 | 0.27 |
| UNPD224655 | (E)-3-((2S,3R)-2-(4-hydroxy-3-methoxyphenyl)-3-(hydroxymethyl)-7-methoxy-2,3-dihydrobenzofuran-5-yl)acrylaldehyde | -22.10 | -28 | 0.26 |
| UNPD230014 | (E)-3-(4-((2S,3R)-2,3-dihydroxy-3-(4-hydroxy-3-methoxyphenyl)propoxy)-3-methoxyphenyl)acrylaldehyde | -21.10 | -39 | 0.35 |
| UNPD224603 | 4-((2S,3S)-3-(hydroxymethyl)-5-((E)-3-hydroxyprop-1-en-1-yl)-7-methoxy-2,3-dihydrobenzofuran-2-yl)-2-methoxyphenol | -20.71 | -23 | 0.21 |
| UNPD151821 | (E)-N-(4-(2-formyl-5-hydroxy-1H-pyrrol-1-yl)butyl)-3-(4-hydroxy-3-methoxyphenyl)acrylamide | -18.33 | -31 | 0.28 |
| UNPD101160 | (1S)-1-((5R)-5-(2-hydroxypropan-2-yl)-2-methyltetrahydrofuran-2-yl)-4-((2R,4aR,6S,8aS)-6-((2S,5S)-5-(2-hydroxypropan-2-yl)-2-methyltetrahydrofuran-2-yl)-8a-methyloctahydropyrano[3,2-b]pyran-2-yl)pent-4-en-1-ol | -17.52 | -31 | 0.20 |
| UNPD202303 | (Z)-3-((R)-5-((1S,4R,E)-1-hydroxy-2,4-dimethyldec-2-en-1-yl)-2-oxodihydrofuran-3(2H)-ylidene)propanoic acid | -15.25 | -28 | 0.28 |


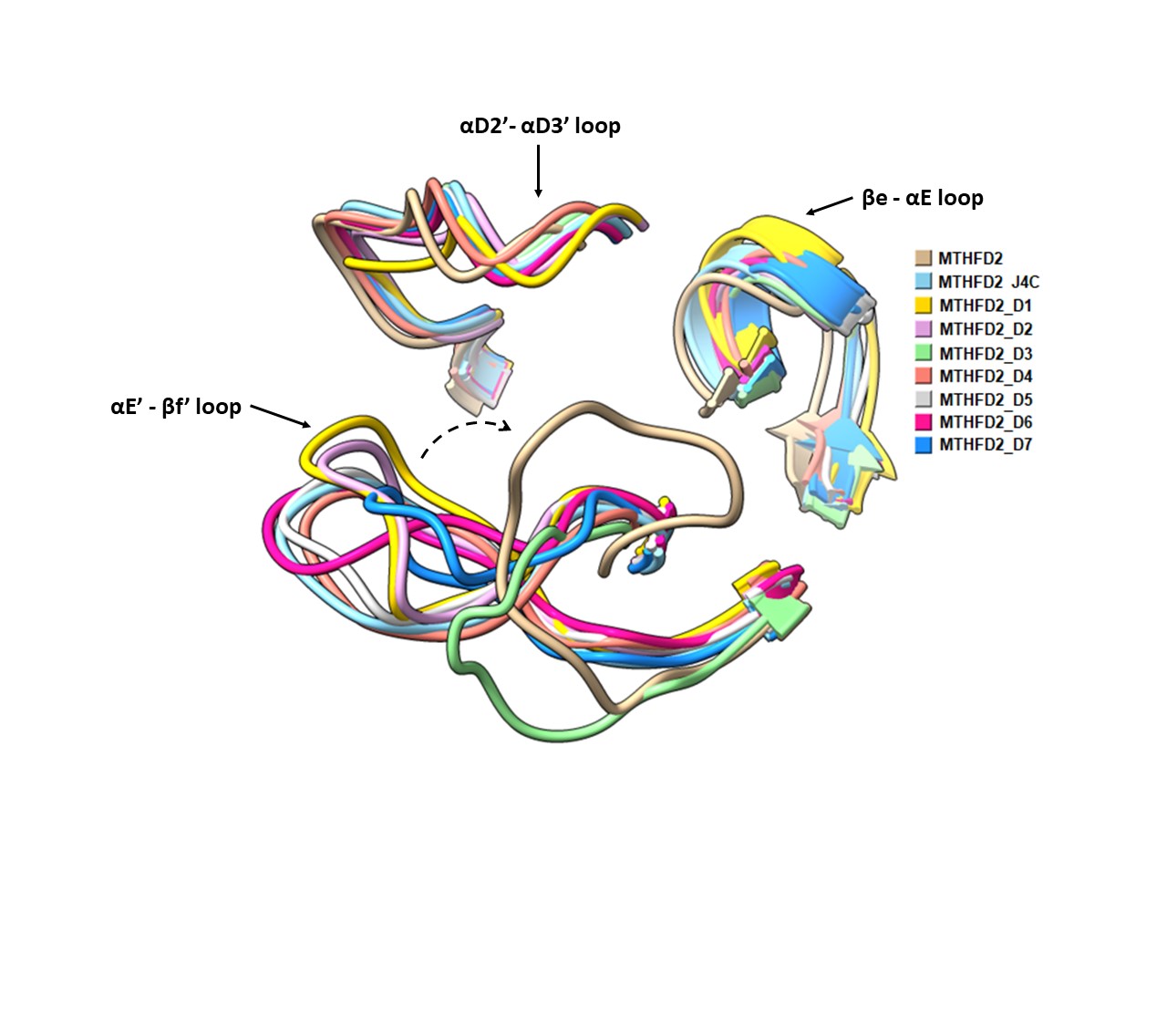


**Supplementary Figure 4:** Superimposed average structures from MTHFD2 docked complex displaying three loops βe-αE, αD2’- αD3’ and αE’-βf’. The highest displacement was observed in αE’-βf’ of all three loops in the presence of J4C reference ligand at the allosteric site in all docked complexes.
